# Tyrosine nitration: An emerging mechanistic landscape in neurodegeneration and cardiopulmonary disease

**DOI:** 10.1016/j.redox.2026.104423

**Published:** 2026-10-03

**Authors:** Nirbachita Adrita, Inge Claassen, Miranda Huerta, Stephen M. Black, Alvaro G. Estevez, Blaine R. Roberts, Joseph S. Beckman, Maria Clara Franco

**Affiliations:** a Center for Translational Science, Florida International University, Port St. Lucie, Florida, 34987, USA; b Department of Cellular & Molecular Medicine, Herbert Wertheim College of Medicine, Florida International University, Port St. Lucie, Florida, 34987, USA; c Department of Biochemistry and Biophysics, Oregon State University, Corvallis, OR, 97331, USA; d Department of Neurology, Emory University School of Medicine, Atlanta, GA, 30322, USA; e Department of Biochemistry, Emory University School of Medicine, Atlanta, GA, 30322, USA; f Linus Pauling Institute, Oregon State University, Corvallis, OR, 97331, USA

**Keywords:** Neurodegeneration, Cardiovascular disease, Pulmonary disease, Reactive nitrogen species, Peroxynitrite, Tyrosine nitration

## Abstract

Protein tyrosine nitration is a post-translational modification associated with conditions of oxidative and inflammatory stress, driven by the production of reactive oxygen and nitrogen species (ROS and RNS). Once considered primarily a footprint of oxidative damage, tyrosine nitration is increasingly recognized as a selective modification that, in specific biological contexts, can alter protein structure, stability, enzymatic activity, and interaction networks. Mechanistically, nitration occurs through peroxynitrite-dependent and heme-peroxidase-dependent pathways and preferentially targets specific tyrosine residues based on local protein structure, solvent accessibility, and microenvironmental chemistry.

Accumulating evidence has identified disease-specific nitration signatures across multiple pathological contexts. In neurodegenerative disorders, including Alzheimer's disease, Parkinson's disease, and amyotrophic lateral sclerosis, nitration of proteins such as tau, α-synuclein, manganese superoxide dismutase, ATP synthase, glyceraldehyde-3-phosphate dehydrogenase, heat shock proteins, and cytoskeletal components contributes to mitochondrial dysfunction, protein aggregation, metabolic failure, and neuronal loss. Similarly, in cardiovascular and pulmonary diseases, protein tyrosine nitration disrupts vascular and epithelial homeostasis by impairing endothelial signaling, fibrinolysis, lipid metabolism, and barrier integrity through modification of key regulatory proteins.

By influencing phosphorylation-dependent signaling, enzymatic function, and protein-protein interactions, tyrosine nitration contributes to the connection between redox imbalance and progressive cellular and tissue dysfunction. This review summarizes current mechanistic insights into site-specific protein tyrosine nitration, highlights the emerging disease-associated nitroproteome, and discusses how advances in analytical and structural approaches are redefining nitration as a functional regulatory modification. Understanding the molecular determinants and consequences of tyrosine nitration may enable the development of targeted diagnostics and therapeutic strategies across diverse human diseases.

## 1 Introduction

Reactive oxygen species (ROS) and reactive nitrogen species (RNS) are central components of redox biology, functioning as essential mediators of physiological signaling while also contributing to pathology and cellular damage when produced at higher levels. ROS such as superoxide (O_2_^•-^) and hydrogen peroxide (H_2_O_2_), together with RNS including nitric oxide (^•^NO) and its downstream oxidant peroxynitrite (ONOO^−^), maintain cellular homeostasis under controlled conditions but promote oxidative, nitrosative, and nitrative stress when dysregulated. ^•^NO synthesis by nitric oxide synthases supports vascular tone regulation, neurotransmission, immune defense, and developmental processes, and its reactivity with oxygen- and metal-derived oxidants shapes the balance between signaling and cytotoxicity [1,2].

One relevant outcome of ROS/RNS interplay is the generation of peroxynitrite and other sources of nitrating species that drive protein tyrosine nitration, a post-translational modification that can serve as both a biomarker and a mediator of oxidative stress [[3], [4], [5], [6]]. Proteins carrying 3-nitrotyrosine accumulate in tissues under conditions where ^•^NO interacts with oxidants such as O_2_•^-^, H_2_O_2_, or metal-containing enzymes. This modification reflects a shift from normal ^•^NO signaling toward oxidative pathways and is strongly associated with inflammation and disease progression [7]. Peroxynitrite and its secondary radicals, including nitrogen dioxide (^•^NO_2_) and carbonate radical (CO_3_^•-^), represent potent biological nitrating species capable of modifying proteins, lipids, and DNA under pathological conditions [1,2,8,9].

In the context of cellular processes, tyrosine nitration can alter the structure and function of critical proteins across multiple organ systems. The functional consequences of nitration are context-dependent, as modification of specific proteins and residues can alter processes involved in cellular signaling and homeostasis [[10], [11], [12], [13], [14]]. These modifications have been linked to multiple diseases including cancer [15], neurodegenerative conditions like Parkinson's and Alzheimer's disease, coronary artery disease, and ischemia-reperfusion injury, positioning nitration as both a disease biomarker and a potential therapeutic target [1,7,10,16].

In neurological diseases, nitration affects mitochondrial proteins, metabolic enzymes, and structural components essential for neuronal survival. Accumulation of nitrated manganese superoxide dismutase (MnSOD), nitrated cytochrome *c*, and other modified mitochondrial proteins impairs respiration, destabilizes mitochondrial membranes, and promotes apoptosis in models of Alzheimer's disease, Parkinson's disease, and ALS [6,10,16].

In the cardiovascular system, protein nitration has been associated with endothelial dysfunction, inflammation, and atherosclerosis. High peroxidase activity and nitrite (NO_2_^−^) oxidation within atherosclerotic plaques further promote nitration and oxidative stress in these regions [1,5,7,17]. In addition, nitration has been reported to impair antioxidant enzymes and reduce ^•^NO availability, changes that contribute to hypertension and reperfusion injury [1,18,19].

Protein tyrosine nitration has also been reported in several pulmonary diseases. Inflammatory airway conditions such as asthma, chronic obstructive pulmonary disease (COPD), and acute respiratory distress syndrome (ARDS), are associated with nitric oxide-derived reactive species and nitrated proteins [20,21].

Recent large-scale proteomic analyses have substantially advanced our understanding of protein tyrosine nitration by systematically mapping its distribution across the human proteome. A recent comprehensive study compiled a curated database of 908 nitrated human proteins encompassing 1971 distinct nitration sites, providing a large-scale view of the reported breadth and selectivity of this modification [13].

Building on three decades of foundational work and recent advances in redox biology, this review delineates how protein tyrosine nitration can alter the function of selected proteins across neurological, cardiovascular, and pulmonary diseases. This framework enables the identification of recurring mechanistic themes and disease-specific vulnerabilities shaped by tyrosine nitration and may help identify nitrated proteins or nitration-associated pathways that warrant further investigation as potential therapeutic targets. Protein tyrosine nitration is increasingly recognized as a selective redox-dependent modification that, in certain biological contexts, may contribute to altered protein function and disease-associated cellular dysfunction. Its presence across cardiovascular, neurological, and pulmonary diseases supports its value as a biomarker of nitrative stress, while mechanistic studies suggest that selected nitration events may also contribute to cellular dysfunction. Understanding how nitration arises, its selectivity, and how it shapes disease progression provides essential insight into redox-driven pathology and informs therapeutic strategies aimed at modulating nitrative stress.

## 2 Mechanism of protein tyrosine nitration

As detailed in Fig. 1, the chemical routes leading to tyrosine nitration involve both non-enzymatic and enzymatic pathways, each involving distinct nitrating species, enzymatic involvement, and biological contexts. The reaction of ^•^NO with O2^•-^ produces peroxynitrite, which rapidly reacts with carbon dioxide (CO_2_) in biological environments to form nitrosoperoxycarbonate. This intermediate decomposes to generate ^•^NO_2_ and CO_3_^•-^, radicals that can participate in protein tyrosine nitration. The one-electron oxidation of a tyrosine residue by CO_3_^•-^ yields a tyrosyl radical, which then reacts with •NO_2_ to form 3-nitrotyrosine (Fig. 1A).

**Fig. 1:**
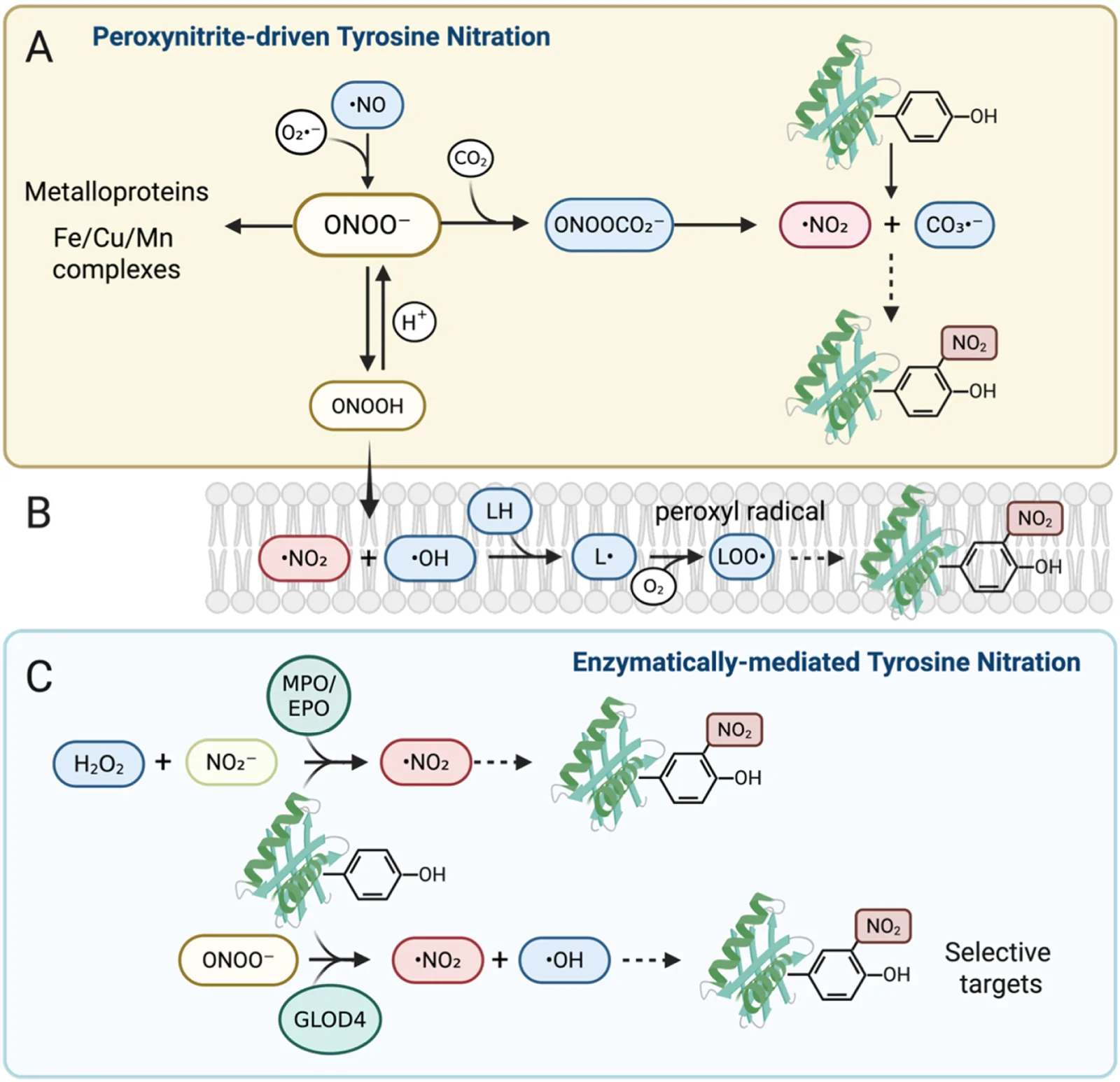
**Chemical and enzymatic mechanisms of protein tyrosine nitration.** **(A)** Nitric oxide (·NO) and superoxide (O_2_·^-^) react to form peroxynitrite (ONOO^−^), which can undergo protonation or react with CO_2_ to generate secondary reactive species, including nitrogen dioxide (·NO_2_) and carbonate radical (CO_3_·^-^). These intermediates promote tyrosyl radical formation and subsequent nitration of protein tyrosine residues. Metal-containing proteins (Fe-, Cu-, and Mn-complexes) can further modulate peroxynitrite reactivity. **(B)** In the plasma membrane, peroxynitrous acid decomposes into derived radicals (·NO_2_) and hydroxyl radical, (·OH) initiating lipid peroxidation chain reactions that generate lipid radicals (L·), and lipid peroxyl radicals (LOO·) that facilitate tyrosine nitration of membrane proteins. **(C)** Inflammatory peroxidases such as myeloperoxidase (MPO) and eosinophil peroxidase (EPO) generate ·NO_2_ from H_2_O_2_ and nitrite (NO_2_^−^), promoting protein nitration. Also, the zinc-dependent enzyme GLOD4 catalyzes peroxynitrite-driven nitration of specific protein substrates. Together, these pathways illustrate both non-enzymatic and enzyme-driven mechanisms that regulate protein nitration under pathological conditions.

A key determinant of which biomolecules will undergo reactions with peroxynitrite, or derived species is compartment chemistry; peroxynitrite chemistry can occur in the aqueous phase (e.g., thiols, CO_2_, metalloproteins), but the protonated form (ONOOH; pK_a_ ∼6.8) can freely diffuse across lipid membranes, enabling secondary radical chemistry in the hydrophobic phase. After ONOOH undergoes homolysis, the resulting radicals, hydroxyl radical (•OH) and •NO_2_, can initiate lipid oxidation by one-electron abstraction of allylic hydrogen atoms from unsaturated fatty acids. In simple chain terms, hydroxyl radical (often described as “OH-like” reactivity) abstracts H from a membrane lipid (LH) to form a lipid radical (L^•^), which rapidly reacts with molecular oxygen (O_2_) to form lipid peroxyl radicals (LOO^•^). These peroxyl radicals can then react with tyrosine residues in membrane proteins to generate a tyrosyl radical that will produce nitrotyrosine by its reaction with ^•^NO_2_ [22] (Fig. 1B). In parallel, reactions involving ^•^NO and ^•^NO_2_ with lipids can generate oxidized and nitrated fatty-acid products, linking nitrative stress to membrane damage and signaling-active lipid species [8,9,22].

In addition, enzymes such as heme peroxidases including myeloperoxidase (MPO) and eosinophil peroxidase (EPO) oxidize NO_2_^−^ to ^•^NO_2_, enabling nitration independently of peroxynitrite formation [8,23]. Quantitation of nitrated proteins in MPO/EPO knockout models of acute inflammation by mass spectrometry revealed a context-dependent role: in neutrophil- and eosinophil-rich tissues such as the vascular wall and pulmonary airways, MPO/EPO appeared to contribute significantly to nitration, most notably of extracellular proteins [24,25], whereas in other inflammatory settings with elevated nitration levels, no such contribution was observed [25].

Importantly, the extent and biological significance of tyrosine nitration depend on the local biochemical environment of a given tissue. Formation of peroxynitrite requires the spatial and temporal overlap of ^•^NO and O_2_^•-^ production [1,8,9,26]. In addition, substrate availability, including l-arginine for NOS-dependent ^•^NO synthesis and nitrite availability for peroxidase-dependent ^•^NO2 generation, may further influence the extent of nitration in different tissues [23,26,27]. In the nervous system, mitochondrial and cellular sources of O_2_^•−^ can coexist with neuronal or inflammation-associated ^•^NO production, creating localized conditions that favor peroxynitrite formation and protein nitration [1,26]. In contrast, in pulmonary and cardiovascular tissues, inflammatory-cell-derived peroxidases, particularly MPO and EPO, can use nitrite to generate nitrating species, thereby contributing to protein tyrosine nitration in inflammatory microenvironments [[23], [24], [25]]. Therefore, tyrosine nitration is strongly influenced by tissue- and disease-specific biochemical conditions.

Nitric oxide synthases (NOS) are central determinants of local ^•^NO availability and therefore strongly influence the formation of nitrating species across disease contexts. Mammalian cells express three major NOS isoforms: neuronal NOS (nNOS/NOS1), endothelial NOS (eNOS/NOS3), and inducible NOS (iNOS/NOS2). eNOS and nNOS are generally regulated by calcium-calmodulin signaling and produce transient, spatially restricted ^•^NO signals, whereas iNOS is transcriptionally induced during inflammatory activation and binds calmodulin with high affinity, allowing more sustained ^•^NO production [27,28]. All NOS isoforms require l-arginine and molecular oxygen as substrates and NADPH, FAD, FMN, heme, and tetrahydrobiopterin (BH4) as cofactors for coupled ^•^NO synthesis [[27], [28], [29]]. In the nervous system, nNOS-derived ^•^NO can react with mitochondrial or NADPH oxidase-derived O_2_^•-^ under conditions of neuronal stress, favoring localized peroxynitrite formation near synaptic, cytoskeletal, and mitochondrial targets [26]. In cardiovascular disease, eNOS normally supports vascular ^•^NO signaling, but reduced l-arginine or BH4 availability, increased asymmetric dimethylarginine (ADMA), oxidative post-translational modifications, or altered regulatory interactions such as reduced Hsp90 coupling can uncouple eNOS, shifting electron flow toward O_2_^•-^ rather than ^•^NO production [26,27,29]. This mechanism is particularly relevant in pulmonary vascular disease, where eNOS uncoupling, BH4 oxidation, and inflammatory induction of iNOS can increase the local coexistence of ^•^NO and O_2_^•-^, favoring peroxynitrite formation and downstream nitration of proteins involved in vascular tone, barrier integrity, and remodeling [30,31]. Thus, the mechanisms that predominantly drive tyrosine nitration in a given organ, and the resulting levels of nitrated proteins, are shaped by disease-specific factors. These include the local sources of O_2_^•-^, the regulation of ^•^NO synthesis through NOS isoform expression, calcium signaling, substrate/cofactor availability, as well as the levels of antioxidants and antioxidant enzymes that set the steady-state concentrations of the reactive species involved. The spatial and temporal compartmentalization of these processes further determines where and when specific proteins may become nitrated.

Adding a new layer to the mechanisms of tyrosine nitration, Wright et al. recently identified GLOD4 as a protein that can enhance selective peroxynitrite-dependent tyrosine nitration and proposed it as a candidate “nitrase”. As described in more detail below in the context of Parkinson's disease, Glyoxalase domain-containing protein 4 (GLOD4) catalyzes peroxynitrite-driven tyrosine nitration of selective targets, including α-synuclein [32]. Unlike MPO and EPO, GLOD4 does not catalyze tyrosine nitration in the presence of H_2_O_2_ and NO_2_^−^ but rather seems to accelerate peroxynitrite decomposition (Fig. 1C).

Table 1 summarizes the key features of the two major pathways involved in generating nitrating agents [1,8,9,17]:

**Table 1:** Major tyrosine nitration pathways.

Pathway	Peroxynitrite (ONOO^−^)	Myeloperoxidase (MPO)/Eosinophil Peroxidase (EPO)-Dependent
Primary Nitration species	Nitrogen dioxide (^•^NO_2_) and carbonate radical (CO_3_^•-^)	^•^NO_2_ and heme peroxidase compounds I and II
Formation/Mechanism	**Non-Enzymatic:** Rapid, diffusion-limited reaction between nitric oxide (^•^NO) and superoxide (O_2_^•-^) to form peroxynitrite (ONOO^−^). ONOO^−^ then reacts with carbon dioxide (CO_2_) to generate the final nitrating radicals.	**Enzymatically mediated:** Heme peroxidases (MPO/EPO) react with H_2_O_2_ forming the intermediate heme peroxidase compounds I and II that mediate oxidation of tyrosine to tyrosyl radical and NO_2_^−^ to ^•^NO_2_.
Biological Location and Context	Primary cellular mechanism. Occurs where ^•^NO and O_2_^•-^ production colocalize.	Primarily at inflammatory sites, driven by activated neutrophils (MPO) and eosinophils (EPO).

Peroxynitrite is not only a precursor of free-radical nitration chemistry; it is also consumed through direct reactions with transition-metal centers. Peroxynitrite can react with iron, copper, or manganese present in metalloproteins or low molecular weight complexes, and these reactions can proceed through one-electron pathways (often yielding NO_2_^−^, e.g., with heme proteins) or two-electron pathways that generate ^•^NO_2_ together with an oxidized metal center. Some metal complexes can also catalyze peroxynitrite isomerization to nitrate (NO_3_^−^), effectively diverting peroxynitrite away from radical-mediated damage. Notably, in heme enzymes such as MPO, peroxynitrite reacts with the heme iron through a one-electron process to produce ^•^NO_2_ and an oxo-ferryl intermediate, which is itself a strong oxidant. In the presence of reductants (e.g., glutathione or ascorbate), these oxidizing intermediates can be reduced back to NO_2_^−^ and the basal metal state [9]. The reaction of MPO with peroxynitrite can therefore also contribute to tyrosine nitration under appropriate biological conditions [1].

## 3 Factors determining the site-specificity of protein tyrosine nitration

Tyrosine nitration generally affects only a small fraction of the total protein pool, and its functional impact may depend on whether the modification occurs at a critical site, within a specific subcellular compartment, or together with other oxidative modifications [33]. A longstanding paradox concerning nitrated proteins *in vivo* is why only a discrete number of proteins and specific residues within those proteins are selectively nitrated when peroxynitrite is endogenously produced by cells in pathological conditions. We and others confirmed such selectivity via the fairly short list of nitrated proteins that have been consistently identified by mass spectrometry (MS) in several disease states and tissues [1,[3], [4], [5], [6],[34], [35], [36], [37]]. The paradox arises because in cells, nitration is most commonly initiated by non-enzymatic oxidation by free radicals from peroxynitrite decomposition. These free radicals attack proteins at many sites, but certain tyrosine residues are consistently more susceptible to nitration [38]. The basis for such selectivity follows a principle established by radiation chemists: initial radical-induced damage to a protein by any strong oxidant is frequently repaired internally by certain tyrosine residues that rapidly reduce the original amino-acid radical via electron transfer and then become tyrosyl radicals [[39], [40], [41]]. The local environment of selective tyrosine positions allows these residues to produce exceptionally stable radicals that can persist for hours [39,42,43]. These persistent tyrosyl radicals react with ^•^NO_2_ produced by peroxynitrite and thereby are prone to become selectively nitrated, producing reproducible nitration patterns in pathological conditions.

Structural features play a central role in determining the selectivity of tyrosine nitration [44,45]. Although no definitive consensus sequence predicts nitration, the local amino acid context strongly influences susceptibility. Tyrosine residues flanked by acidic amino acids, particularly glutamate, are more frequently nitrated. Nitration is further favored when tyrosines are positioned within ∼3 Å of a negatively charged residue and reside in microenvironments that lack cysteine and methionine. The relative paucity of nitration near cysteine residues has traditionally been attributed to the higher reactivity of RNS with thiols; however, this paradigm is not absolute in cellular contexts. For example, peroxiredoxin 2 displays nitrotyrosine formation in close proximity to cysteine residues, with a minimal Tyr-Cys distance of 3.6 Å (PDB: 1QMV) [46,47]. In addition, tyrosines located within five residues of proline or glycine exhibit a higher incidence of modification, likely because these residues introduce bends or turns that increase tyrosine exposure within flexible loop regions. Beyond primary sequence, protein tertiary structure further constrains nitration, which typically occurs on surface-exposed tyrosines, although not all accessible residues are modified [44]. Initial studies on the selectivity of tyrosine nitration also showed that the nature of the nitrating pathway, such as peroxynitrite-dependent *versus* MPO/H_2_O_2_/NO_2_^−^ -dependent nitration, may influence which proteins are preferentially targeted in different contexts [45].

Fulfillment of these requirements provides two layers of specificity to nitrotyrosine location: 1) only a subset of proteins will ever contain nitrotyrosine, and 2) only some tyrosines within that subset of proteins will become nitrated. In addition, another level of selectivity may be provided by enzymes able to catalyze nitration of specific tyrosine residues, such as peroxidases, metal-containing oxidoreductases, and hemoproteins. For example, SOD-catalyzed nitration of specific tyrosine residues in NF-L by peroxynitrite reflects selectivity inherent to the structural features of NF-L itself [38,48]. It is tempting to speculate on the existence of selective cellular nitrases that modify tyrosine residues within specific targets, an area that has started to emerge, with GLOD4 reported to increase the propensity of α-synuclein nitration, though the mechanistic basis of this effect warrants further investigation [32].

In addition to the specificity underlying nitrotyrosine formation, the selective nature of protein tyrosine nitration determined by the positional context of the modified tyrosine and its local structural environment raises the possibility that it may serve as a biologically meaningful post-translational modification in certain contexts [38]. Unlike phosphorylation, which can occur dynamically and is enzymatically reversible, tyrosine nitration typically affects only a small fraction of the total protein pool and represents a relatively stable covalent modification that may persist until protein turnover [38,49]. This stability may generate sustained alterations in protein function that contribute to pathological states rather than serving as a reversible regulatory switch [49]. In some cases, tyrosine nitration promotes ubiquitination or proteasomal degradation. This has been reported for HDAC2, in which nitration of Y253 contributes to proteasome-dependent loss under nitrative stress [50], an effect that can, in turn, have consequences for cellular signaling. Thus, whether nitration ultimately alters protein function and downstream signaling depends not only on protein abundance but also on how nitration affects protein half-life and turnover. Several studies have also reported loss of nitrotyrosine immunoreactivity or apparent reversal of nitration in selected systems, including histone H1.2, calmodulin, and KGDHC, with the reaction products examined in some cases by mass spectrometry [[51], [52], [53]]. Still, whether the nitro group is truly removed to regenerate tyrosine or instead reduced to aminotyrosine remains uncertain. Because the changes observed may also reflect antibody recognition, chemical reduction of the nitro group, protein turnover, or substrate-specific repair processes, and because the denitrase has not been purified, the molecular identity, substrate range, and generality of a mammalian denitrase that removes the nitro group remain to be established.

Although peroxynitrite and 3-nitrotyrosine are most commonly discussed in the setting of oxidative stress, selective protein nitration can also be detected during normal physiological processes in restricted contexts. In rat embryonic heart development, nitrotyrosine immunoreactivity shows a striking temporal pattern: strong staining during mid-gestation (embryonic days 12-17, E12–E17) that markedly declines by late gestation (E18) and disappears at birth [54]. These nitration events are spatially confined predominantly in trabeculae and atria and are largely associated with terminally differentiated cardiomyocytes rather than proliferating or apoptotic zones, suggesting a regulated association with differentiation/remodeling programs rather than nonspecific cell death [54]. Proteomic identification of nitrated targets in embryonic hearts includes key contractile proteins (α-actin and β-myosin heavy chain) and an ATP synthase related target, indicating that nitration can occur on functional structural/metabolic proteins during development [54]. Together, these data support the idea that peroxynitrite-derived nitration is not exclusively pathological but can appear transiently in specific physiological windows, such as organ development, whereas sustained or widespread nitration is more characteristic of disease-associated nitrative stress. Notably, even outside overt disease settings, nitration can have structural consequences, as shown by NO_2_^−^-mediated non-enzymatic nitration of collagen, which produces cross-linking and structural modifications resembling age-related tissue damage [55].

Protein tyrosine nitration is particularly relevant in the context of human disease, where selected nitration events have been linked to altered enzymatic activity, signaling pathways, and cellular dysfunction. Across neurodegenerative, cardiovascular, and pulmonary disorders, elevated nitrative stress creates a biochemical environment that favors nitration of proteins with structurally permissive features, including surface-exposed tyrosines, nearby acidic residues, flexible loop regions, or local microenvironments with limited competing cysteine and methionine residues [44,45]. Many of these proteins play important roles in metabolism, cytoskeletal integrity, mitochondrial function, and inflammation [13]. In addition, tyrosine nitration may also affect immune recognition of self-proteins. Introduction of 3-nitrotyrosine can modify existing antigenic determinants or generate neoepitopes, potentially increasing the immunogenicity of otherwise self-proteins. Immune responses against nitrated self-proteins have been reported in inflammatory and autoimmune settings, suggesting an additional biological consequence of nitrative stress [56].

Studies show that disease-specific nitration signatures correlate with severity, tissue vulnerability, and progression [6,7]. Here, we focused on proteins that when nitrated, show strong evidence for functional disruption in human disease. These proteins emerged repeatedly across independent datasets, mechanistic studies, and pathological tissues, indicating that their nitration is both biologically meaningful and clinically relevant [13]. The following sections focus on these prominent targets and outline how site-specific nitration reshapes their structure, signaling roles, and contributions to disease progression in neurodegenerative conditions, and in cardiopulmonary diseases. A table at the end summarizes site-specific tyrosine nitration events across these diseases, indicating for each protein the functional effect of nitration, the experimental system used, and the extent of *in vivo* evidence. It further compiles other oxidative modifications reported for the same proteins, not necessarily studied in the same disease context, highlighting the potential for interplay if such modifications were to coincide with nitration on shared protein targets.

## 4 Role of nitrated proteins in neurodegenerative diseases

Neurons are uniquely vulnerable to protein nitration due to their high metabolic demand, reliance on mitochondrial respiration, and limited capacity for protein turnover. Elevated production of O_2_^•-^ and ^•^NO in neuronal mitochondria creates a biological setting that favors peroxynitrite formation, driving selective nitration of proteins involved in energy metabolism, cytoskeletal stability, and neurotransmission. Nitrated proteins, including MnSOD, α-synuclein, tau, and components of the ATP synthase complex have been consistently identified in Alzheimer's disease (AD), Parkinson's disease (PD), and amyotrophic lateral sclerosis (ALS), where their modification contributes to mitochondrial failure, impaired proteostasis, synaptic dysfunction, and neuronal death [6,10,16]. In these disorders, tyrosine nitration functions as a biomarker of oxidative stress, while selected nitration events may contribute to neurodegenerative mechanisms.

### 4.1 Alzheimer's disease

Alzheimer's disease (AD) is characterized by an insidious onset with progressive deterioration of cognitive and behavioral functions. AD accounts for approximately 60–80% of dementia cases in individuals aged 65 years and older, affecting an estimated 7.2 million Americans [57]. Pathologically, AD is defined by two hallmark lesions: (i) extracellular deposition of amyloid-β (Aβ) peptides forming senile plaques in the brain, and (ii) intracellular accumulation of hyperphosphorylated tau protein as neurofibrillary tangles (NFTs) [58]. Aβ peptides isolated from the brains of patients with AD exhibit multiple post-translational modifications [59,60]. Among these, protein tyrosine nitration is a frequently observed oxidative modification and has been reported to alter the function of several essential proteins, including numerous critical targets nitrated in AD brain tissue [61,62].

#### 4.1.1 Effect of tau tyrosine nitration on microtubule formation and aggregation

Tau is a microtubule-associated protein (MAP) encoded on chromosome 17, and alternative mRNA splicing generates six isoforms expressed in the adult human brain [[63], [64], [65]]. In AD, all isoforms can be found within neuropil threads, neuritic plaques, and neurofibrillary tangles (NFTs), which are hallmark pathological inclusions that strongly correlate with neuronal dysfunction and cognitive decline [66]. Interest in tyrosine nitration grew substantially once this modification was identified in tau and in proteins linked to multiple neurodegenerative disorders [10,67].

Tau primary sequence contains five tyrosine residues, Y18, Y29, Y197, Y310, and Y394. Studies *in vitro* showed that nitration occurs selectively at Y18, Y29, Y197, and Y394, all located in the N-terminal and proline-rich domains of the protein [68]. *In situ* immunohistochemical analyses in AD brain tissue using site-specific antibodies further revealed site-specific nitration (nY) patterns: Tau-nY18 labeling colocalizes primarily with glial pathology, whereas Tau-nY29 is enriched in neuronal NFTs and dystrophic neurites [69,70] (Fig. 2).

**Fig. 2:**
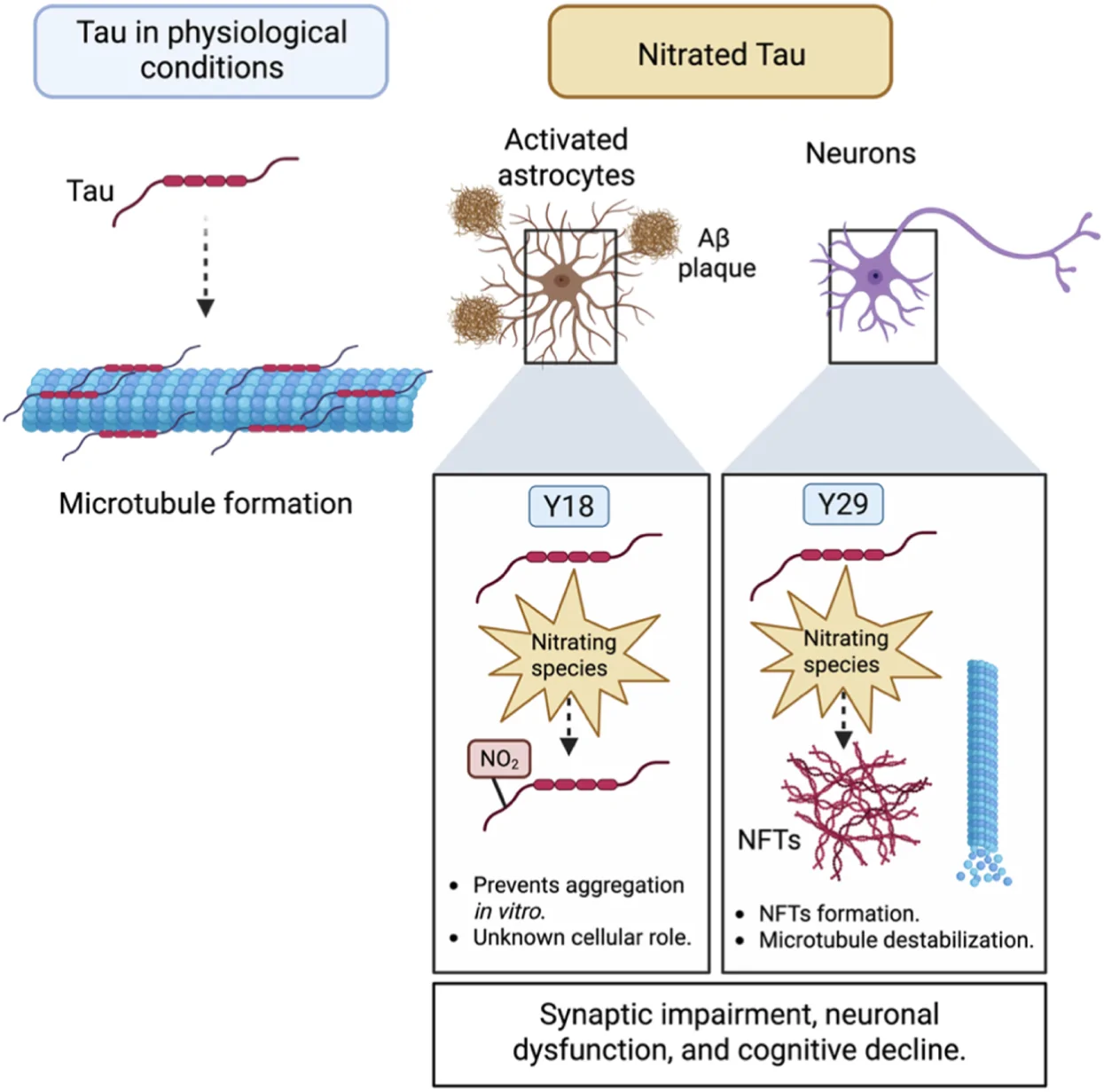
**Functional consequences of tau tyrosine nitration.** Under physiological conditions, tau binds to and stabilizes microtubules, supporting axonal structure and intracellular transport. In contrast, tau nitration alters its structural and functional properties. Nitration at distinct tyrosine residues exerts site-specific effects. Nitration at Y18 is detected in astrocytes and has been reported to prevent tau aggregation *in vitro*, although its precise cellular role remains unclear. Conversely, nitration at Y29 promotes the formation of neurofibrillary tangles (NFTs) and contributes to microtubule destabilization in neurons. These modifications shift tau from a microtubule-stabilizing protein to a pathological species associated with cytoskeletal disruption. Collectively, tau nitration contributes to synaptic impairment, neuronal dysfunction, and progressive cognitive decline.

These observations indicate that tau nitration is not random but follows isoform and cell type specific patterns, suggesting a regulated and potentially pathogenic process. The functional consequences of tau nitration may contribute to AD pathology. Nitration of tau impairs its microtubule-binding ability, destabilizes axonal microtubules, and promotes tau self-aggregation, thereby promoting the formation of neurofibrillary tangles [[70], [71], [72]]. Because tau is essential for maintaining axonal transport, its nitration-mediated dysfunction may disrupt the trafficking of vesicles, mitochondria, and signaling molecules along the axon. Through these mechanisms, tyrosine nitration transforms tau from a stabilizing cytoskeletal protein into a toxic driver of synaptic impairment, neuronal dysfunction, and cognitive decline.

#### 4.1.2 Effect of Aβ tyrosine nitration on aggregation, toxicity, and synaptic dysfunction

Beta-amyloid (Aβ) is a peptide that arises from the proteolytic processing of the amyloid precursor protein (APP) and is central to the pathophysiology of Alzheimer's disease (AD) [73,74]. Aβ exists in various forms, with Aβ1-42 being particularly prone to aggregation into plaques that are characteristic of AD [75,76]. The amyloid cascade hypothesis postulates that the early formation of soluble Aβ is a critical initiating event leading to neuronal damage and cognitive decline in AD [74]. Besides being a hallmark of the disease, Aβ is thought to play roles in normal physiological processes, such as neuroprotection and synaptic function [75,77]. As AD progresses, the accumulation of Aβ contributes to neuroinflammation and disrupts neural signaling, driving further degeneration [78,79]. Nitration of Aβ, particularly at Y10, has been linked to pathological outcomes in AD, as this modification stabilizes oligomeric forms of the peptide, promoting the accumulation of highly reactive assemblies. These oligomeric species enhance N-methyl-d-aspartate receptor (NMDAR)-mediated excitotoxicity, contributing to neuronal injury and loss of synaptic integrity [80]. Thus, rather than favorably modulating fibril formation, Y10 nitration promotes the stabilization of toxic oligomeric assemblies and disrupts neuronal signaling homeostasis, thereby exacerbating synaptic dysfunction (Fig. 3).

**Fig. 3:**
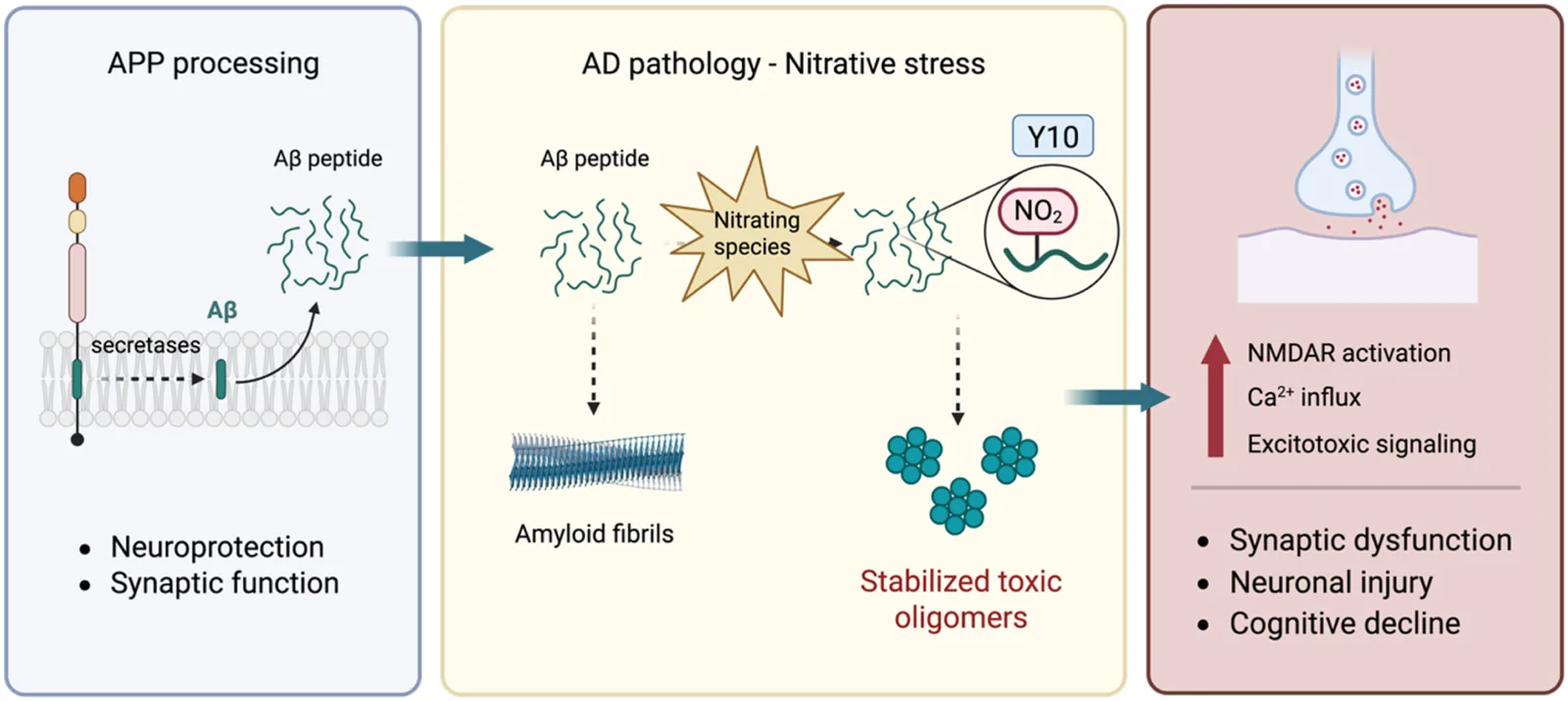
**Physiological and pathological roles of amyloid-β (Aβ) and the impact of tyrosine nitration in Alzheimer's disease (AD).** Under physiological conditions, amyloid precursor protein (APP) is processed by secretases to generate amyloid-β (Aβ) peptides, which participate in neuroprotection and synaptic function. In AD, nitrative stress promotes tyrosine nitration at Y10 in Aβ. This modification alters Aβ aggregation behavior, shifting it from fibrillar assembly toward the stabilization of toxic oligomeric species. Nitrated Aβ oligomers enhance N-methyl-d-aspartate receptor (NMDAR) activation, leading to increased Ca^2+^ influx and excitotoxic signaling. These events contribute to synaptic dysfunction, neuronal injury, and progressive cognitive decline.

The presence of 3-nitrotyrosine has also been observed in the brain of AD patients, reinforcing its role as a biomarker for pathological conditions associated with amyloid pathology [81]. Under pathophysiological conditions, interactions between amyloid-β (Aβ) and reactive nitrogen species lead to increased levels of peptide nitration, which correlate with disease severity [82]. This interplay highlights the need for deeper investigation into the underlying biochemical pathways and how they might be leveraged to inform therapeutic development in Alzheimer's disease. In summary, tyrosine nitration of Aβ represents a potentially important modification in AD pathogenesis, influencing both peptide aggregation and neurotoxicity.

#### 4.1.3 ATP synthase α-subunit tyrosine nitration impairs mitochondrial energy production

The ATP synthase α-subunit is a core component of the F_1_ catalytic head of mitochondrial ATP synthase, forming part of the α_3_β_3_ hexamer that is essential for ATP production. Although the α-subunit binds ATP, it is not catalytically active; instead, it stabilizes the catalytic β-subunits and supports rotational coupling during ATP synthesis. Disruption of ATP synthase function, including aberrant cytoplasmic accumulation of the α-subunit, has been reported in AD, linking altered subcellular localization of this subunit to mitochondrial dysfunction and impaired cellular energy metabolism [83].

Tyrosine nitration of ATP synthase, particularly its alpha subunit, has been identified as a significant modification in the context of AD. ATP synthase plays a crucial role in cellular energy production through oxidative phosphorylation in mitochondria, and its subunits undergo substantial post-translational modifications under conditions of oxidative stress, which can impair ATP production [84,85]. In AD pathology, oxidative and nitrative stressors can induce nitration of ATP synthase and potentially disrupt its normal function, critical for neuronal health [84,86]. This impairment may contribute to reduced ATP levels in neurons, subsequently impacting essential processes such as ion transport and synaptic function, which are vital for maintaining neuronal integrity [86]. Furthermore, Aβ has been reported to interact with ATP synthase, particularly the OSCP subunit, contributing to mitochondrial and synaptic dysfunction in AD [87,88]. Protein nitration may also affect ATP synthase interactions with other proteins and metabolic pathways.

This complex interplay underscores the multifaceted role of ATP synthase dysfunction in AD, highlighting the need for further investigations into targeted therapeutic strategies that can mitigate nitrative stress and restore normal ATP synthase functionality [89]. In redox proteomic datasets, Y337 and Y440 of the ATP synthase α subunit (ATP5F1A) have been identified as potential sites of nitration [13]. While the direct functional consequences of these specific modifications remain uncharacterized, their location within the catalytic F_1_ domain suggests that nitration could impair ATP production by disrupting conformational changes required for substrate binding and phosphorylation. Given that ATP synthase function is tightly regulated by structural integrity and protein-protein interactions, selective nitration at these sites may contribute to the mitochondrial dysfunction commonly observed in AD. Tyrosine nitration of the ATP synthase alpha subunit in AD reflects the intersection of oxidative stress, mitochondrial dysfunction, and impaired neuronal energy metabolism, although the direct consequences of specific nitration sites remain to be fully defined.

#### 4.1.4 Tyrosine nitration of GAPDH drives metabolic dysfunction and protein aggregation

Glyceraldehyde-3-phosphate dehydrogenase (GAPDH) is a key enzyme in the glycolytic pathway, catalyzing the conversion of glyceraldehyde-3-phosphate to 1,3-bisphosphoglycerate while facilitating NAD^+^ reduction to NADH [90]. Beyond its metabolic role, GAPDH is also involved in various cellular processes, including redox balance, signal transduction, and apoptosis [[90], [91], [92]]. Notably, in the context of Alzheimer's disease (AD), GAPDH has been shown to interact with amyloid-beta (Aβ) peptides, forming cytotoxic complexes that may contribute to neurodegeneration [91,93].

Tyrosine nitration of GAPDH has been identified in AD brain, and GAPDH is susceptible to modification under peroxynitrite-mediated nitrative stress [62,94]. This post-translational modification can lead to the formation of aberrant protein aggregates and affect the enzyme's normal function, disrupting glycolysis and contributing to metabolic dysregulation in neuronal cells. Notably, GAPDH has been reported to interact with Aβ and to promote Aβ aggregation and cytotoxicity in AD-related models [91,93]. Consequently, understanding the implications of GAPDH nitration in AD could provide insights into potential therapeutic targets aimed at mitigating neuronal loss and cognitive decline in affected patients.

GAPDH has been identified as a nitrated protein in human AD hippocampus [62]. Separately, proteomic studies performed in other biological and experimental contexts have identified Y140, Y276, Y314, and Y320 as potential sites of GAPDH tyrosine nitration [13,[94], [95], [96], [97]]. The functional consequences of nitration at these individual residues remain unknown; however, given their location within functionally relevant regions of GAPDH, such modifications could potentially influence catalytic activity, protein interactions, localization, or stability.

#### 4.1.5 Effect of tyrosine nitration on Hsp70 chaperone activity

Hsp70 is a 70-kDa stress-induced molecular chaperone that supports neuronal survival by maintaining proteostasis, preventing protein misfolding, and reducing aggregation of amyloid-β and tau. In AD, Hsp70 expression increases in glial cells and neurons during early neurodegenerative stress, and its overexpression reduces neuronal injury in multiple experimental models [98,99].

Nitration of Hsp70 in different contexts has been described. However, the pathological consequences remain to be determined. Under nitrative stress, MS analysis of peroxynitrite-treated PC12 cells identified Hsp70 as one of the predominant endogenous proteins that become tyrosine-nitrated alongside tubulin, actin, and Hsp90 [34]. Importantly, when nitrated Hsp70 was delivered intracellularly to PC12 cells and motor neurons, it did not induce cell death, unlike nitrated Hsp90 which gained a toxic function, as discussed in detail below. These findings indicate that Hsp70 nitration occurs in disease-relevant oxidative environments but potentially does not directly trigger cytotoxicity. Instead, based on the known effects of tyrosine nitration in other proteins such as impaired enzymatic activity, disrupted signaling, and altered structural stability documented across multiple nitrated proteins [100], nitration of Hsp70 in AD may weaken its chaperone efficiency. Such impairment could diminish its ability to prevent aggregation of pathogenic Aβ and tau, thereby compounding proteostasis failure characteristic of the AD brain.

Hsp70 nitration at Y336 was identified in an MS analysis dataset [13]. This residue is located within the substrate-binding domain of Hsp70, and nitration could reduce substrate affinity, impair the allosteric communication between the nucleotide-binding and substrate-binding domains, and disrupt interactions with co-chaperones that regulate ATP-dependent folding cycles. Given that nitrated proteins typically exhibit loss-of-function rather than toxic gain-of-function behavior (with Hsp90 being a notable exception), nitration at Y336 is most consistent with diminished chaperone capacity. In the context of AD, where Hsp70 plays a critical protective role in suppressing Aβ and tau aggregation, loss of function caused by Y336 nitration would be expected to compromise proteostasis and further promote the accumulation of neurotoxic protein species.

#### 4.1.6 MnSOD tyrosine nitration affects the mitochondrial antioxidant balance

Manganese superoxide dismutase (MnSOD, also known as SOD2) is a key mitochondrial antioxidant enzyme localized primarily within the matrix, where it catalyzes the dismutation of O_2_^•-^ into H_2_O_2_ and molecular oxygen (O_2_), thereby limiting oxidative stress and preserving mitochondrial redox homeostasis [[101], [102], [103], [104]]. In the context of AD, MnSOD tyrosine nitration represents a disease-associated post-translational modification that can compromise enzymatic activity and antioxidant function.

Nitration of MnSOD at Y34 has been associated with reduced enzymatic activity and impaired antioxidant function [18,105]. The specificity is provided by the rapid initial reaction of peroxynitrite with the Mn center [106]. This modification not only reduces the dismutation efficiency of O_2_^•-^ but may also lead to the formation of dysfunctional aggregates that exacerbate mitochondrial dysfunction, a hallmark of neurodegeneration in AD [102,107]. Nitration of MnSOD correlates with decreased antioxidant defense capabilities in neurons, potentially worsening the cellular environment and promoting the progression of AD [101,107]. Additionally, tyrosine nitration of MnSOD has been associated with decreased MnSOD activity and impaired mitochondrial respiration, which can weaken mitochondrial antioxidant defense and contribute to oxidative/nitrative stress in AD pathology [107] (Fig. 4). The resulting decrease in MnSOD activity can contribute to a feedforward loop of oxidative damage, emphasizing the importance of maintaining the functional integrity of this enzyme in combating disease-related oxidative stress [102,105]. Understanding the mechanisms and consequences of MnSOD tyrosine nitration may provide insights into novel therapeutic strategies aimed at enhancing mitochondrial function and redox balance in AD patients.

**Fig. 4:**
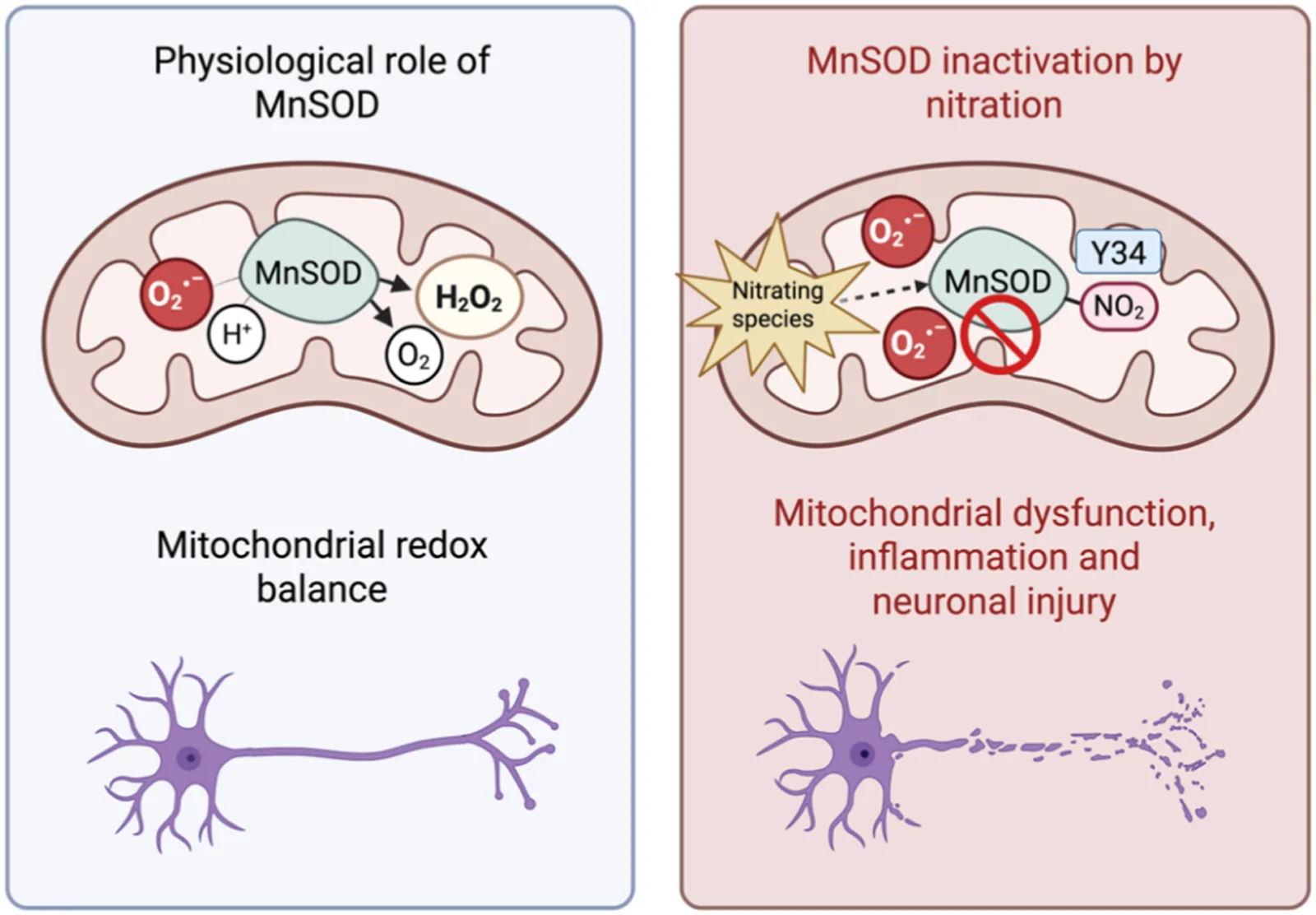
**Physiological function of MnSOD and its inactivation by tyrosine nitration in neurodegeneration.** Under physiological conditions, mitochondrial manganese superoxide dismutase (MnSOD) catalyzes the dismutation of superoxide (O_2_•^-^) into hydrogen peroxide (H_2_O_2_) and molecular oxygen (O_2_), thereby maintaining mitochondrial redox balance and supporting neuronal viability. Under conditions of increased nitrative stress, site-specific nitration of MnSOD at Y34 is associated with reduced enzymatic activity. Loss of MnSOD activity leads to mitochondrial oxidative imbalance, enhanced inflammatory signaling, and accumulation of oxidative damage, ultimately contributing to neuronal dysfunction and injury.

### 4.2 Amyotrophic lateral sclerosis

Amyotrophic lateral sclerosis (ALS) is a progressive neurodegenerative disease characterized by the degeneration of both upper and lower motor neurons in the brain and spinal cord, leading to muscle weakness, atrophy, spasticity, and eventual respiratory failure, typically within a few years of diagnosis [108]. Although the precise pathogenic mechanisms remain incompletely understood, mutations in numerous genes have been identified as causative contributors to ALS, including the gene encoding Cu,Zn-superoxide dismutase (SOD1), one of the first and best-characterized ALS genes [109,110]. While sporadic ALS accounts for approximately 90% of cases, genetic analyses frequently reveal mutations in ALS-associated genes even in patients without a clear family history. Current evidence supports a multifactorial etiology in which genetic susceptibility, environmental exposures, and age-related biological changes collectively drive disease onset and progression. Neuropathologically, ALS is defined by progressive degeneration of the motor system and the spread of pathological changes through interconnected neural networks, often beginning focally and advancing to anatomically and functionally related regions [111].

ALS is marked by profound oxidative and nitrative stress resulting from mitochondrial dysfunction, misfolded proteins, and elevated production of O_2_^•-^ and ^•^NO, creating the biochemical conditions that favor tyrosine nitration and other damaging post-translational modifications [112]. Tyrosine nitration is increasingly recognized as a pathological feature of amyotrophic lateral sclerosis (ALS). Studies have shown that 3-nitrotyrosine accumulates in ALS tissues, indicating widespread nitrative stress in degenerating motor neurons. An important source of this modification is peroxynitrite. In ALS models, oxidative stress promotes peroxynitrite formation, which in turn triggers inflammatory signaling. Together, these findings support a role for tyrosine nitration in mitochondrial dysfunction, inflammation, and progressive motor neuron injury in ALS [34,[113], [114], [115], [116]].

#### 4.2.1 Tyrosine nitration of neurofilament light polypeptide (NF-L)

Neurofilament-light chain (NF-L) is a key cytoskeletal intermediate filament protein in motor neurons, essential for maintaining axonal structure, caliber, and transport. In ALS, NF-L is one of the earliest and most consistently altered proteins, with both sporadic and familial cases demonstrating massive neurofilament aggregation in motor neurons, forming axonal spheroids and perikaryal conglomerates that reflect underlying cytoskeletal dysfunction [117]. These NF aggregates often co-localize with markers of oxidative and nitrative stress, including SOD1 and nitrotyrosine, suggesting that NF-L is exposed to damaging reactive nitrogen species in the ALS spinal cord [117,118]. The vulnerability of NF-L to nitration is also supported by biochemical studies showing that neurofilament proteins are robust substrates for peroxynitrite-mediated nitration and that nitrated NF-L accumulates in ALS spinal cords, contributing to abnormal filament assembly and axonal transport defects [48,119]. Nitration of NF-L at Y17, Y138, Y177, and Y265 was identified following ONOO^−^ treatment *in vitro* in the presence of SOD1 and correlated with decreased neurofilament assembly [48]. Importantly, elevated free nitrotyrosine, an indicator of widespread protein nitration, appears early in ALS-like disease in SOD1 mutant mice, coinciding with the first detectable neurofilament abnormalities even before overt motor neuron loss [120].

MS analyses identified Y372 on human NF-L as an additional site prone to nitration in the cellular context [13,97]. Y372 lies within the C-terminal region of NF-L, a domain essential for lateral spacing of neurofilaments, interactions with NF-M/NF-H, and the formation of stable 10-nm filaments. Nitration at this position would be expected to introduce steric hindrance, disrupt hydrogen bonding, and interfere with the electrostatic interactions required for proper filament cross-bridging. Because this region directly contributes to filament stoichiometry and coiled-coil stabilization, modification of Y372 could impair NF assembly, reduce filament stability, or promote aggregation effects consistent with the neurofilament disorganization observed in both sporadic and SOD1-linked ALS. Moreover, nitration of a residue in this structural “hotspot” could prevent normal phosphorylation–dephosphorylation cycles within the tail domain, further exacerbating transport defects and accelerating the formation of pathological inclusions. Thus, Y372 nitration likely represents a mechanistically meaningful event that links oxidative/nitrative stress to structural collapse of the axonal cytoskeleton in ALS.

#### 4.2.2 Tyrosine Nitration of Hsp90 and its functional consequences in ALS

Heat shock protein 90 (Hsp90) is an essential, ATP-dependent molecular chaperone that is highly abundant in eukaryotic cells (∼1-2% of total cellular protein under non-stress conditions) and promotes the folding, stabilization, and functional maturation of a large client network (often cited as >300 proteins) that spans survival signaling, metabolic control, mitochondrial homeostasis, and stress adaptation [121]. Under physiological conditions, cytosolic Hsp90α (inducible) and Hsp90β (constitutive) maintain proteostasis, repress aberrant signaling, and protect cells from stress. However, five tyrosine residues in Hsp90 are targets for nitration (Y33, Y56, Y276, Y484, Y596) [34]. Among these, Y33 and Y56 are residues that when nitrated drive pathologies of the nervous system. Nitration of a single tyrosine residue on Hsp90 is sufficient to induce motor neuron death, and Hsp90 nitrated at Y56 (Hsp90_NY56_) is endogenously present in spinal motor neurons from human ALS patients and the G93A mouse model [34], establishing this modification as a pathological feature of ALS-related neurodegeneration. Recently, accumulation of Hsp90_NY56_ in degenerating spinal motor neurons was also identified in an ALS-related mouse model of human mutant dynactin subunit 1 (DCTN1) [122]. Mechanistically, nitrated Hsp90 activates the purinergic receptor and calcium channel P2X7, leading to a calcium influx, and finally Fas-dependent apoptosis, a death pathway strongly implicated in ALS motor neuron degeneration [34].

Notably, as shown in Fig. 5, nitrated Hsp90 seems to play different roles depending on the cell type. Hsp90NY56 consistently drives apoptosis in both motor neurons and the neuronal PC12 cell model [123]. On the other hand, nitration at Y33 (Hsp90NY33) induces motor neuron death but is not toxic to PC12 cells, where it localizes to mitochondria, forming a complex that downregulates mitochondrial membrane potential, oxygen consumption, and ATP production, without inducing cytochrome *c* release, revealing a metabolic dimension to Hsp90 nitration [123,124]. These metabolic effects are highly relevant to ALS, where impaired oxidative phosphorylation and energetic failure are central features of motor neuron vulnerability. Collectively, these studies demonstrate that nitrated Hsp90 acts as a molecular switch that converts a normally pro-survival chaperone into a driver of ALS-relevant motor neuron death (Fig. 5). Through P2X7-dependent apoptotic signaling and mitochondrial dysfunction, nitrated Hsp90 links oxidative stress to the selective vulnerability of motor neurons. The presence of endogenously nitrated Hsp90 in human ALS motor neurons and its ability to reproduce key aspects of ALS pathology, including impaired survival pathways, mitochondrial defects, and Fas-mediated apoptosis, strongly position Hsp90 nitration as a mechanistic contributor to motor neuron loss in ALS. Interestingly, in Schwann cells, nitrated Hsp90 acts as a metabolic switch to induce cell proliferation by a mechanism that is also driven by P2X7R. Hsp90NY56 increases glycolysis, and Hsp90NY33 downregulates mitochondrial metabolism [35] (Fig. 5). Proliferation of a subpopulation of Schwann cells has been reported in ALS [125], highlighting the contribution of nitrated Hsp90 to different aspects of the disease process.

A critical question arising from these observations is how nitrated Hsp90 acquires distinct novel functions. Structural studies demonstrate that nitration at Y33 and/or Y56 significantly impacts Hsp90 structure. Nitration at Y33 induces destabilization of the Hsp90 dimer, leading to formation of stable oligomeric species, while nitration at Y56 leads to an altered dimer conformation, which may underpin the pathological gain-of-function. Notably, at least *in vitro*, nitration at Y56 facilitates nitration at Y33, adding a layer of regulation to Hsp90 nitration [121] (Fig. 5).

**Fig. 5:**
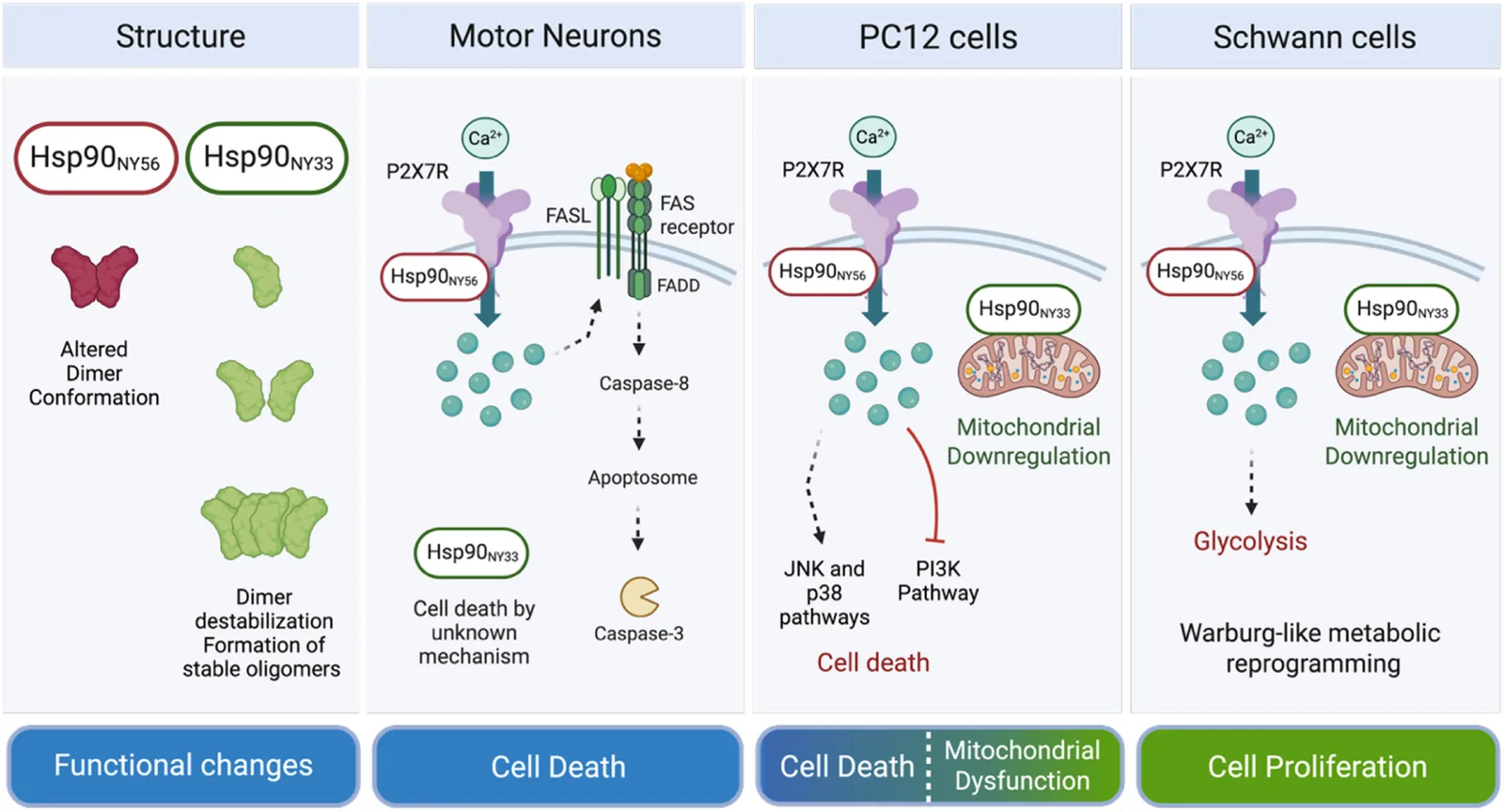
**Cell type-specific functional consequences of Hsp90 tyrosine nitration.** Tyrosine nitration of Hsp90 at distinct residues induces conformational and functional changes with divergent cellular outcomes. **Structure:** Nitration at Y56 (Hsp90_NY56_) alters dimer conformation, whereas nitration at Y33 (Hsp90_NY33_) promotes dimer destabilization and the formation of stable oligomers. **Motor neurons:** Hsp90_NY56_ is associated with P2X7R activation and engagement of extrinsic apoptotic signaling through FAS/FASL, FADD, caspase-8, and caspase-3, leading to cell death. In contrast, Hsp90_NY33_-mediated cell death may occur through a yet undefined mechanism. **PC12 cells:** Hsp90_NY56_ promotes P2X7R-dependent signaling, mitochondrial, activation of stress-associated pathways (JNK and p38), and inactivation of PI3K pro-survival signaling, culminating in cell death. Hsp90_NY33_ contributes to mitochondrial downregulation without inducing cell death. **Schwann cells:** In this context, Hsp90_NY56_ drives P2X7R activation and increased glycolysis, and Hsp90_NY33_ downregulates the mitochondria, promoting a Warburg-like metabolic reprogramming and cell proliferation rather than death. Collectively, these findings illustrate how site-specific nitration of Hsp90 elicits distinct structural alterations that translate into cell type-dependent outcomes, including functional changes, mitochondrial dysfunction, apoptosis, or proliferative metabolic rewiring.

### 4.3 Parkinson's disease

Parkinson's disease (PD) is a progressive neurodegenerative disorder primarily characterized by the loss of dopaminergic neurons in the substantia nigra, a brain region crucial for movement control. The pathophysiology of PD involves the accumulation of misfolded proteins, particularly α-synuclein, which aggregates to form Lewy bodies and Lewy neurites, hallmark features of the disease [126,127]. Clinically, PD manifests with motor symptoms such as tremor, rigidity, bradykinesia, and postural instability, alongside non-motor symptoms including cognitive impairment, depression, and autonomic dysfunction [127]. As an age-related disorder, the incidence of PD is rising in parallel with increasing life expectancy, posing significant global healthcare challenges [128].

Tyrosine nitration is a reported feature of oxidative and nitrative stress in PD, reflecting the increased production of reactive nitrogen species in dopaminergic neurons [129,130]. Peroxynitrite-mediated nitration modifies important neuronal proteins and alters their stability and turnover. Elevated levels of 3-nitrotyrosine have been consistently observed in the substantia nigra of PD patients, indicating widespread protein nitration in affected regions [131,132]. Several proteins essential for neuronal function undergo this modification in PD, including α-synuclein and tyrosine hydroxylase, both of which are found in their nitrated forms [[133], [134], [135]]. Nitration is also detected in additional cellular proteins involved in neuronal homeostasis, contributing to impaired cellular resilience under oxidative stress. This post-translational modification accumulates alongside other markers of oxidative injury and is strongly associated with the degeneration of dopaminergic neurons [129,130]. Overall, tyrosine nitration serves as both a biochemical hallmark and a contributor to neuronal vulnerability in PD.

#### 4.3.1 Tyrosine nitrated alpha-synuclein in Parkinson's disease pathogenesis

Alpha-synuclein (α-syn) is a small, presynaptic protein of approximately 140 amino acids that plays a crucial role in synaptic function and neurotransmitter release. Its significance becomes prominently apparent in the context of neurodegenerative diseases, particularly Parkinson's disease (PD), where it is implicated in the formation of Lewy bodies, intracellular aggregates that are characteristic of the disease pathology. Mutations and multiplications of the α-synuclein gene (*SNCA*) have been linked to familial forms of PD [127,136]. While the normal physiological function of α-synuclein includes regulating synaptic vesicle trafficking and maintaining synaptic integrity, its propensity to misfold and aggregate can lead to detrimental effects on neuronal health, contributing to dopaminergic neuron degeneration [7].

Tyrosine nitration of α-synuclein affects its structure and function (Fig. 6). Key tyrosine residues reported to undergo nitration in α-synuclein include Y39, Y125, Y133, and Y136 [137,138]. Nitration of α-synuclein can enhance its aggregation propensity, leading to accelerated fibrillation and promoting the formation of toxic oligomers, which are implicated in neuronal cytotoxicity [126]. The pathological consequences of tyrosine nitration in α-synuclein are significant. Nitrated forms of α-synuclein are more likely to aggregate, and this aggregation is often associated with increased neurotoxicity. Studies have shown that nitrated α-synuclein can disrupt normal cellular functions, leading to impaired synaptic transmission and increased neuronal vulnerability to stress [126]. Moreover, the presence of nitrated α-synuclein has been confirmed in post-mortem brain tissues from patients with PD, suggesting that this modification may contribute to disease progression and pathogenesis [126]. The presence of tyrosine nitration implicates α-synuclein in a broader network of oxidative stress responses that characterize neurodegenerative disorders. It has been observed that nitration can interfere with the inherent functions of α-synuclein and may contribute to mitochondrial dysfunction, exacerbating the neurodegenerative process associated with Parkinson's disease [126]. Therefore, understanding the effects of tyrosine nitration on α-synuclein is crucial for elucidating its role in PD and could provide insights into potential therapeutic approaches aimed at reducing oxidative stress and its detrimental implications in neurodegeneration.

**Fig. 6:**
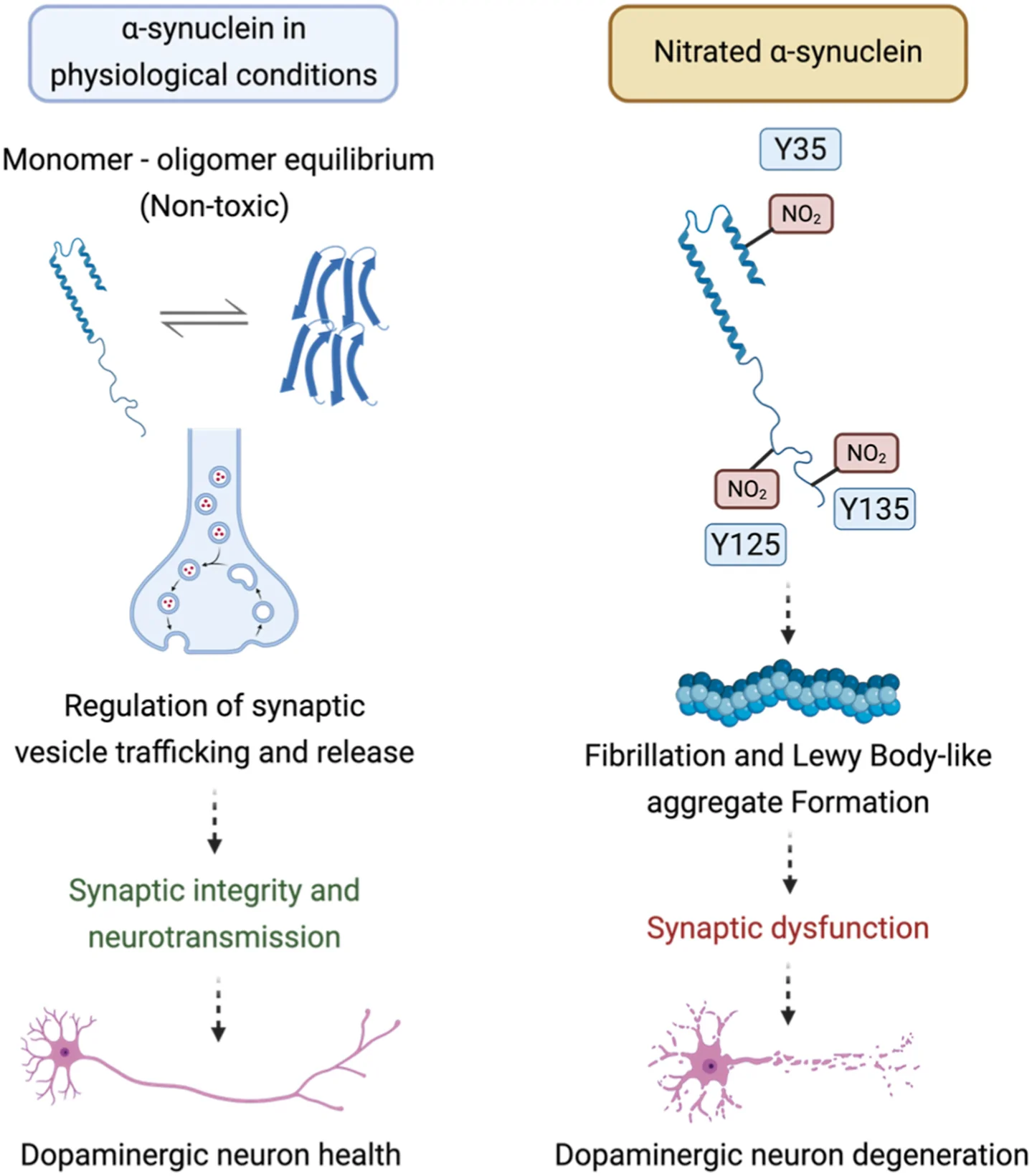
**Physiological and pathological consequences of α-synuclein nitration**. Under physiological conditions, α-synuclein exists predominantly as a soluble, dynamic protein that associates with synaptic vesicles and contributes to synaptic integrity and neurotransmission. In contrast, under conditions of nitrative stress, α-synuclein undergoes site-specific tyrosine nitration, including at residues Y35, Y125, and Y135. These modifications alter protein conformation and promote the formation of oligomeric and aggregated species. Nitrated α-synuclein displays an increased propensity for self-association and reduced normal synaptic function, leading to synaptic dysfunction and neuronal impairment.

Recent evidence suggests that α-syn nitration can occur through enzyme-mediated mechanisms. An unbiased biochemical screen identified GLOD4 as a zinc-dependent enzyme that selectively catalyzes peroxynitrite-driven nitration of α-syn at multiple tyrosine residues (Y39, Y125, Y133, and Y136) *in vitro*. GLOD4-mediated tyrosine nitration of α-syn was also validated *in vivo*, both in the striatum and in peripheral red blood cells of methamphetamine-treated mice [32]. Unlike peroxidases such as MPO and EPO, GLOD4 does not catalyze α-syn nitration in the presence of H_2_O_2_ and NO_2_^−^. Mechanistically, GLOD4 seems to accelerate peroxynitrite decomposition and selectively catalyze tyrosine nitration of α-syn and β-syn. In addition, genetic deletion of GLOD4 markedly reduces basal and inducible α-syn nitration in cells and mouse brain, while pharmacologic inhibition suppresses nitration *in vivo*. Collectively, these findings suggest that α-syn nitration can be a regulated, enzyme-driven process and position GLOD4 as a potential prototype “nitrase,” implicating selective protein nitration as a mechanistic contributor to α-syn–associated neurodegenerative disorders and a potential therapeutic target [32].

#### 4.3.2 Tyrosine nitration of tyrosine hydroxylase and its role in dopamine loss in Parkinson's disease

Tyrosine hydroxylase (TH) is the enzyme responsible for the first and rate-limiting step in catecholamine biosynthesis, converting l-tyrosine into l-DOPA, the precursor of dopamine, norepinephrine, and epinephrine. Because this step determines the overall rate of catecholamine production, TH is essential for normal dopaminergic and noradrenergic function in the brain and peripheral nervous system. TH is primarily expressed in dopaminergic neurons, sympathetic neurons, and the adrenal medulla, where it supports neurotransmission, movement control, stress responses, and other physiological functions regulated by catecholamines [139].

Studies have shown that exposure of TH to peroxynitrite or ^•^NO_2_ induces nitration of specific tyrosine residues and significantly reduces catalytic activity, indicating that this nitrative modification may directly impair dopamine biosynthesis [140]. Mass spectrometric analyses identified Y423, Y428, and Y432 as the principal nitration sites on TH, and modification of these residues disrupts enzymatic function in a region important for structural stability and catalytic efficiency [141]. Because TH is the rate-limiting enzyme required for dopamine synthesis, impaired activity due to nitration at these residues provides a potential mechanistic link between oxidative and nitrative stress and reduced dopamine production. In the context of Parkinson's disease, where dopaminergic neurons are exposed to elevated oxidative stress, TH nitration may contribute to progressive dopaminergic dysfunction.

## 5 Role of nitrated proteins in cardiovascular and pulmonary diseases

In the cardiovascular and pulmonary systems, excessive production of •NO-derived oxidants creates a biochemical milieu that strongly favors tyrosine nitration. Vascular inflammation, endothelial dysfunction, and immune cell activation markedly increase local generation of O_2_^•-^, hydrogen peroxide, and myeloperoxidase (MPO)-dependent radicals, thereby enhancing peroxynitrite formation and nitrotyrosine accumulation in tissues [7]. In the vasculature, this oxidative-nitrative shift disrupts normal endothelial signaling, reduces ^•^NO bioavailability, promotes vasoconstriction, and drives pro-thrombotic remodeling, collectively contributing to hypertension, atherosclerosis, and ischemia-reperfusion injury.

A similar paradigm operates in the pulmonary system. Chronic airway inflammation, as observed in chronic obstructive pulmonary disease (COPD), as well as acute inflammatory insults such as acute respiratory distress syndrome (ARDS) and acute lung injury (ALI), and acute infectious injury, are associated with peroxynitrite production and also robust activation of MPO- and eosinophil peroxidase (EPO)-dependent pathways that generate high levels of ^•^NO_2_. These conditions promote extensive nitration of structural, cytoskeletal, and regulatory proteins within the airway epithelium and pulmonary endothelium [142]. Such modifications compromise barrier integrity, increase vascular permeability, and amplify inflammatory signaling, thereby exacerbating tissue injury and disease progression.

### 5.1 Cardiovascular disease

#### 5.1.1 Tyrosine nitration of fibrinogen as a mediator of prothrombotic risk

Fibrinogen is a key plasma glycoprotein integral to the hemostatic process and the formation of blood clots. It is a large, multi-domain dimeric protein composed of three pairs of polypeptide chains (Aα, Bβ, and γ) that undergoes a complex transformation during coagulation [143,144]. When vascular injury occurs, thrombin cleaves fibrinogen into fibrin monomers, which spontaneously polymerize to form a stable fibrin clot that provides structural integrity to the hemostatic plug and halts bleeding [145,146].

The functionality of fibrinogen can be significantly altered by post-translational modifications, including tyrosine nitration, which can lead to structural and functional changes in fibrinogen. This modification can influence clot formation, stability, and susceptibility to fibrinolysis [147,148]. Notably, tyrosine nitration of fibrinogen has been linked to increased clot density and mechanical strength, suggesting that it may contribute to a prothrombotic state, a condition favoring thrombosis [149]. Structural studies have indicated that nitrated fibrinogen can undergo conformational changes that promote dityrosine crosslinking, which further supports the stability of the resultant fibrin networks [147]. Fibrinogen is a major target of tyrosine nitration under cardiovascular oxidative and nitrative stress. Specific nitration sites have been identified, including Bβ-chain nitration at Y292 and Y422, which alters the biochemical behavior of the fibrinogen molecule, leading to accelerated clot formation, denser and stiffer fibrin networks, and impaired fibrinolysis [150]. Additionally, nitration can occur across the Aα, Bβ, and γ-chains, with modified residues detected in experimental nitration models of fibrinogen (e.g., purified fibrinogen exposed to nitrating oxidants/peroxynitrite-generating conditions), indicating that multiple chains are susceptible under defined nitrative stress conditions [147]. Nitration-driven structural changes disrupt normal fibrin polymerization, reduce clot porosity, promote dityrosine crosslinking or aggregation, and generate fibrin networks with lower mechanical resilience, all of which contribute to a prothrombotic phenotype in cardiovascular disease settings. Collectively, these findings establish nitrated fibrinogen as both a functional mediator of thrombosis and a biomarker of nitrative stress, reflecting its central role in vascular pathology [17].

#### 5.1.2 Tyrosine nitration of plasminogen and fibrinogen: impaired fibrinolysis and prothrombotic consequences

Fibrinogen is a soluble plasma protein that is converted by thrombin into insoluble fibrin, forming the structural scaffold of blood clots during coagulation. In contrast, plasminogen is the inactive precursor of plasmin, the principal protease responsible for fibrinolysis, the regulated dissolution of fibrin clots. Once activated, plasmin degrades fibrin and cleaves fibrinogen and other clotting factors, thereby limiting clot size and preventing excessive thrombosis [[151], [152], [153]]. The tight coordination between coagulation and fibrinolysis ensures that clots form efficiently to arrest bleeding and are subsequently removed to restore normal blood flow [154]. Disruption of this balance, including through oxidative and nitrative modifications of key proteins, can profoundly dysregulate hemostasis. Several studies have reported nitration of fibrinogen, plasminogen, and plasmin in different pathological contexts, which ultimately dysregulates the coagulation process.

Tyrosine nitration of plasmin has been demonstrated *in vivo* and is detectable in human plasma, with elevated levels observed in smokers, a population characterized by increased oxidative and nitrative stress [7]. Nitration reduces plasmin catalytic activity and impairs its proteolytic efficiency toward fibrin substrates, thereby suppressing fibrinolysis, and nitration can interfere with plasminogen activation, further limiting the availability of functional plasmin [155]. Given that plasmin is the primary enzyme responsible for clot dissolution, its nitration promotes a pro-thrombotic state by attenuating fibrinolytic capacity. In cardiovascular disease, plasmin has been identified among plasma proteins susceptible to nitration under conditions of heightened oxidative and nitrative stress [156].

Nonetheless, impaired fibrinolysis in this context appears to be more strongly linked to nitration of fibrinogen, which yields dense, rigid fibrin networks that are resistant to plasmin-mediated lysis [157]. Elevated levels of tyrosine nitration in fibrinogen have also been found *in vivo* and are detectable in human plasma [17,158]. Several tyrosine residues in the Aα, Bβ and γ chains are targets for nitration. The impact of nitration on fibrinogen has been thoroughly addressed in a recent and comprehensive review by Nencini et al., which describes how nitration induces conformational rearrangements, preferentially targeting tyrosine residues within functional regions of the Aα, Bβ, and γ chains [147]. These nitration events affect the tertiary and quaternary structure of fibrinogen, resulting in the formation of high-molecular weight aggregates. The structural alterations disrupt fibrin polymerization and collectively favor a pro-thrombotic state. Given the depth of this prior analysis, we provide only a concise overview here and refer the reader to the cited review for a detailed mechanistic discussion [147].

Consistent with these findings, increased fibrinogen nitration has been reported in several pathological settings. Nitrated fibrinogen serves as a biomarker of oxidative stress in venous thromboembolism [158] and is prominently detected in smokers, with preferential nitration at Y292 and Y422 within the β chains [150]. Elevated nitration has also been observed in patients with cardiovascular disease [158] and acute respiratory distress syndrome [20]. More broadly, nitration at multiple tyrosine residues across all three fibrinogen chains has been documented; however, the specific sites identified *in vitro* and *in vivo* differ, likely reflecting variations in fibrinogen conformation, the influence of other post-translational modifications, and differences in the nitrating species involved. Functionally, peroxynitrite-treated fibrinogen *in vitro* gives rise to fibrin clots that are more deformable yet structurally weaker [159], and in smokers, high circulating levels of nitrated fibrinogen correlate with increased clot stiffness *in vivo* [150].

Collectively, these observations indicate that nitration of fibrinogen and plasminogen alters both the kinetics of fibrin formation and the structural and biomechanical properties of clots, leading to dysfunctional hemostasis and increased thrombotic risk [158,159]. (Fig. 7).

**Fig. 7:**
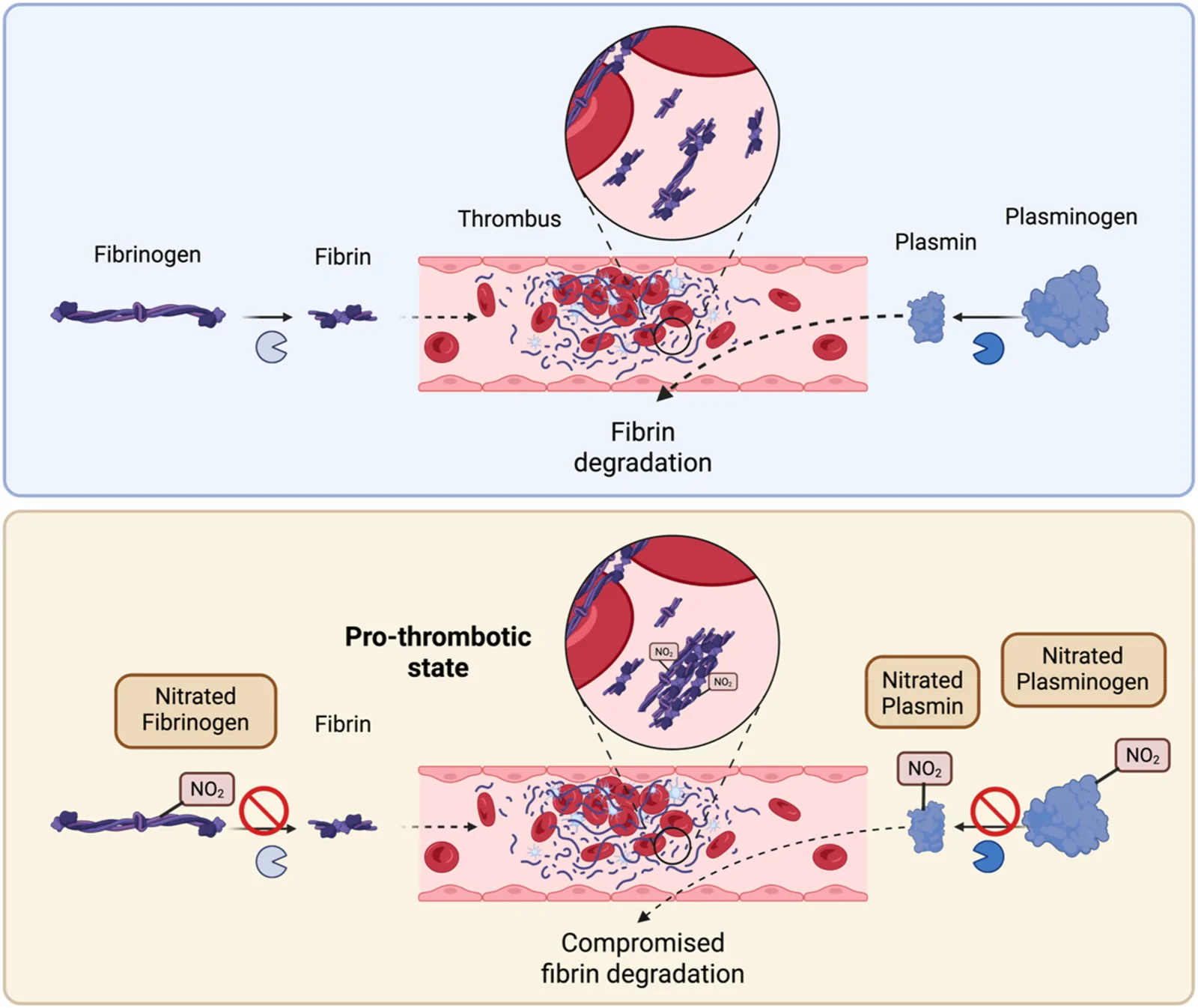
**Impact of tyrosine nitration on fibrin formation and fibrin**olysis. Under physiological conditions (top), fibrinogen is converted to fibrin to form a stable thrombus, which is subsequently resolved through plasmin-mediated fibrinolysis following activation of plasminogen. This coordinated balance between clot formation and degradation ensures effective hemostasis and restoration of blood flow. Under conditions of nitrative stress (bottom), tyrosine nitration of fibrinogen, plasminogen, and plasmin disrupts this balance. Nitrated fibrinogen forms structurally abnormal fibrin networks, while nitration of plasmin and plasminogen reduces plasmin generation and catalytic efficiency, collectively impairing fibrin degradation. These combined effects favor a pro-thrombotic state characterized by persistent clots and compromised fibrinolysis.

#### 5.1.3 Tyrosine nitration of apolipoprotein A-I: site-specific modifications driving HDL dysfunction in atherosclerosis

Apolipoprotein A1 (ApoA-I) is the principal protein component of high-density lipoprotein (HDL), a type of lipoprotein known for its role in reverse cholesterol transport. This process involves the removal of excess cholesterol from peripheral tissues and its subsequent delivery to the liver for excretion. ApoA-I is synthesized primarily in the liver and intestine, playing a crucial role in lipid metabolism and cardiovascular health [160].

ApoA-I performs multiple essential functions. In addition to serving as the principal structural component of high-density lipoprotein (HDL), it facilitates cellular cholesterol efflux through interaction with the membrane transporter ATP-binding cassette transporter A1 (ABCA1), which mediates lipid export from cells [161,162]. This interaction is essential for the biogenesis of HDL particles and their anti-atherogenic properties, which help to prevent the accumulation of cholesterol in the arterial walls and reduce the risk of atherosclerosis [163]. Modifications to ApoA-I, such as nitration and oxidation, can impair its function and thus compromise its protective effects against cardiovascular diseases [163,164]. These modifications can occur in the presence of oxidative stress and inflammation, linking ApoA-I function directly to overall cardiovascular health and disease risk.

As detailed in Fig. 8, tyrosine nitration of apolipoprotein A-I (apoA-I) occurs at specific residues and contributes to dysfunctional HDL in atherosclerosis. In the lipid-free state, Y192 is highly accessible to aqueous oxidants and becomes the major site of nitration, while additional residues including Y18, Y29, Y115, Y166, and Y236 can also be nitrated depending on oxidant exposure in HDL-associated forms [165]. When apoA-I binds lipids, Y192 becomes buried within a more hydrophobic environment, reducing its susceptibility to nitration and shifting nitrative modifications to other solvent-exposed sites [165]. Within human atherosclerotic arteries, Y166 is a prominent physiological nitration site and represents a substantial fraction of total apoA-I present in the artery wall [166]. This modification selectively impairs LCAT activation, leading to reduced catalytic efficiency, while nitration at Y192 minimally affects ABCA1-mediated cholesterol efflux. ApoA-I recovered from atherosclerotic lesions consistently contains nitrated tyrosine residues, demonstrating an oxidative arterial environment enriched in MPO- and ^•^NO-derived reactive species that modify lipoproteins during disease progression [150,166]. Overall, apoA-I nitration is site-specific, influenced by lipid environment, and functionally linked to impaired HDL activity in cardiovascular disease.

**Fig. 8:**
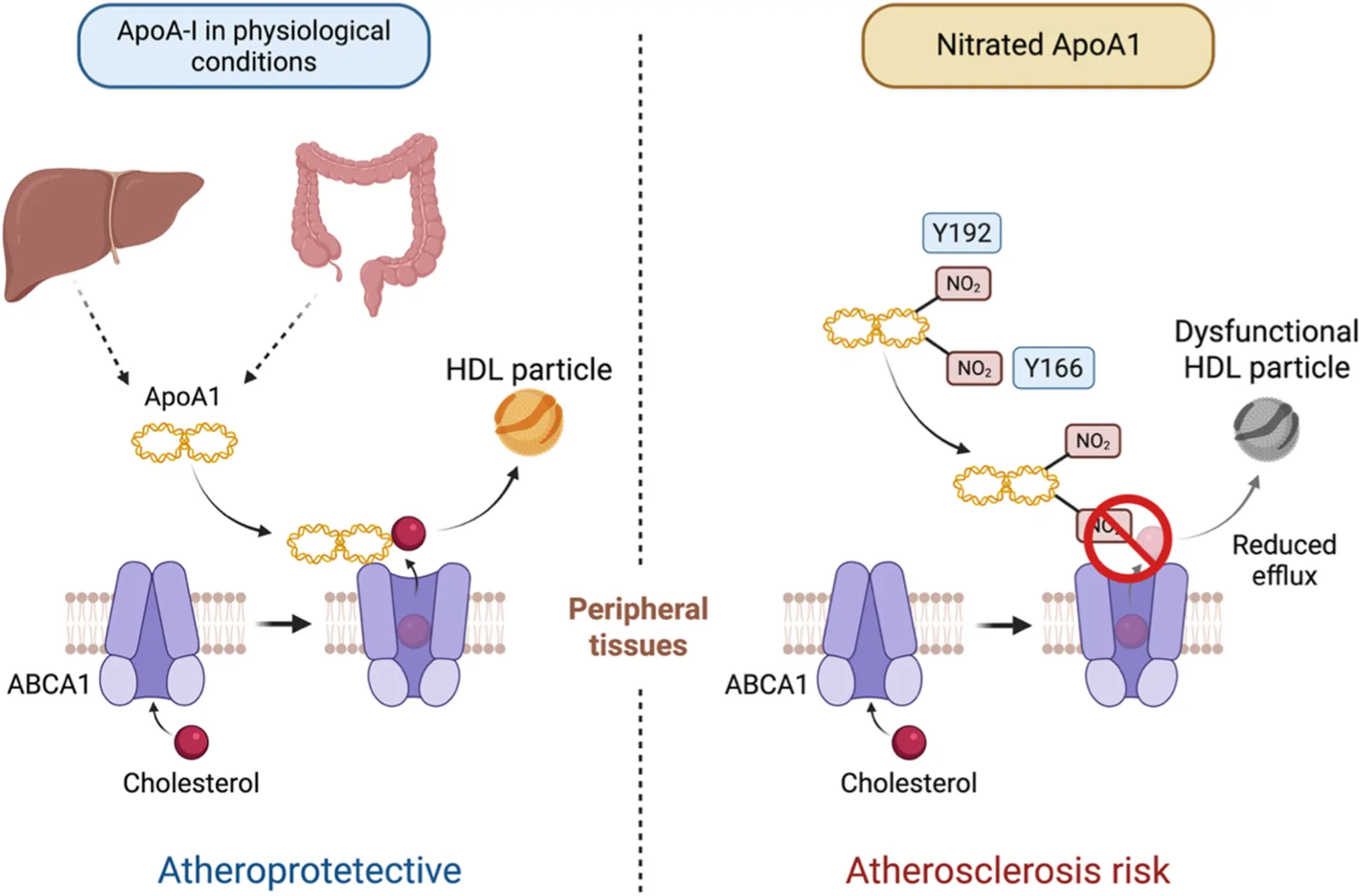
**Functional consequences of ApoA-I nitration in lipid homeostasis and atherosclerosis.** Under physiological conditions, apolipoprotein A-I (ApoA-I) serves as the principal structural and functional component of high-density lipoprotein (HDL), promoting cholesterol efflux from peripheral tissues through interaction with the ATP-binding cassette transporter A1 (ABCA1). In contrast, under conditions of nitrative stress, ApoA-I undergoes site-specific tyrosine nitration, including at residues Y192 and Y166, which impairs its interaction with ABCA1 and diminishes cholesterol efflux capacity. Nitrated ApoA-I compromises HDL functionality, disrupts lipid homeostasis, and contributes to lipid accumulation within peripheral tissues, thereby increasing atherosclerotic risk. This figure illustrates how nitration converts ApoA-I from an atheroprotective mediator to a dysfunctional HDL component that promotes cardiovascular pathology.

#### 5.1.4 Tyrosine nitration of LDL and ApoB-100: structural destabilization and enhanced atherogenicity

Low-density lipoprotein (LDL) is a major lipoprotein class in the human circulatory system that plays a pivotal role in lipid transport and metabolism. Composed primarily of cholesterol and triglycerides, LDL particles primarily deliver cholesterol to peripheral tissues via receptor-mediated endocytosis involving LDL receptors (LDLR) [167]. Cholesterol derived from LDL is critical for maintaining cellular membrane integrity and serving as a precursor for steroid hormones and bile acids [168]. The physiological significance of LDL is underscored by its association with atherosclerosis and cardiovascular diseases. Elevated plasma levels of LDL cholesterol (LDL-C) are recognized as a key risk factor for the development of coronary artery disease, making it a primary target for therapeutic interventions aimed at lowering cardiovascular risk [169]. Biochemically, LDL consists of a single molecule of apolipoprotein B-100 (ApoB-100), which plays a crucial role in the structural integrity and functionality of the lipoprotein. The ratio of apolipoprotein B to ApoA-I is often used as a biomarker for cardiovascular risk assessment, where higher ratios indicate greater atherogenic potential [170]. Furthermore, growing evidence suggests that modifications to LDL, such as oxidation and nitration, can enhance its pathogenicity and contribute to the progression of atherosclerosis [171].

Tyrosine nitration of ApoB-100 is a major oxidative modification detected in atherogenic LDL fractions. *In vivo*-modified LDL (LDL^−^) shows a marked increase in nitrotyrosine content compared with native LDL, demonstrating extensive nitration of ApoB-100 tyrosine residues. LC/MS/MS analysis identifies specific nitrated tyrosines within ApoB-100, including Y276, Y666, Y720, Y2524, Y3139, Y3295, Y3489, and Y4141, confirming that nitration occurs across multiple structural domains of the protein [13]. These nitrated residues lie within α-helical and β-sheet regions, and their modification is associated with significant loss of α-helical structure and a gain in β-sheet/random coil, indicating ApoB-100 unfolding. This unfolding coincides with elevated lipid peroxides in LDL^−^, suggesting combined lipid and protein oxidation drives structural destabilization. Functionally, nitrated LDL shows greater binding and uptake by LOX-1 (lectin-like oxidized LDL receptor-1), CD36 (cluster of differentiation 36/fatty acid translocase), and SR-A (scavenger receptor class A), than native LDL. These are scavenger receptors that bind and internalize oxidized LDL, indicating that tyrosine nitration alters receptor specificity and enhances atherogenic behavior [172]. Notably, pro-inflammatory macrophages take up oxidized LDL via scavenger receptors, driving their transformation into foam cells and accelerating atherosclerotic plaque formation [173,174].

#### 5.1.5 Tyrosine nitration of COX-1: disruption of thromboxane-dependent vascular homeostasis

Cyclooxygenases (COX) are a group of enzymes integral to the conversion of arachidonic acid to prostaglandins and thromboxanes, which are vital mediators of biological activities, including inflammation and vascular homeostasis. There are two main isoforms of COX: constitutive COX-1 and inducible COX-2. COX-1 is constitutively expressed in many tissues and is primarily responsible for the production of prostaglandins that maintain physiological functions, such as protecting the gastric mucosa and regulating platelet function. It plays a crucial role in the maintenance of normal physiological processes, including renal blood flow and gastrointestinal integrity [17,175]. For example, COX-1 converts arachidonic acid into thromboxane A_2_, a potent promoter of platelet aggregation and vasoconstriction. COX-2 expression, on the other hand, is induced in response to inflammatory stimuli. It contributes significantly to the inflammatory response by synthesizing prostaglandins that mediate pain, fever, and inflammation. Elevated COX-2 levels have been linked to various inflammatory diseases and conditions, including atherosclerosis [176].

COX-1 is nitrated and inhibited under defined nitrative stress conditions. Purified holo–PGHS-1 (COX-1) exposed to peroxynitrite *in vitro*, particularly in the absence of arachidonic acid, undergoes nitration at the essential catalytic residue Y385, which results in loss of cyclooxygenase activity [177]. *In vivo*, COX nitration has been detected in human atherosclerotic tissue and in aortic lesions from ApoE^−/−^ mice maintained on a high-fat (atherogenic) diet, and tyrosine nitration in ApoE^−/−^ mice is reported to be iNOS-dependent [178,179]. Overall, tyrosine nitration of COX-1 specifically at Y385 disrupts prostanoid balance and contributes to the pro-thrombotic, pro-inflammatory environment of cardiovascular disease [17]. Interestingly, Y385 is highly conserved within the COX family, suggesting that nitration may also affect activity of other family members, such as COX-2, which could also have pathological consequences. Nitration of COX-2 has been reported in the context of diseases such as diabetic angiopathy [180], and carrageenan-induced thermal hyperalgesia and oedema [181], although the specific nitration sites have not been confirmed.

#### 5.1.6 Tyrosine nitration of SERCA: impaired calcium handling in cardiac muscle dysfunction

The sarco(endo)plasmic reticulum Ca^2+^-ATPase (SERCA) is a vital enzyme responsible for the transport of calcium ions (Ca^2+^) from the cytosol into the sarcoplasmic reticulum (SR), an essential organelle in muscle cells that regulates intracellular calcium levels. By facilitating the removal of Ca^2+^ from the cytoplasm, SERCA plays a critical role in muscle relaxation and overall cellular calcium homeostasis [182,183]. In the cardiac muscle, SERCA2a is particularly important, as it regulates contractility and relaxation of heart muscle cells, making it a crucial component in cardiac physiology [183,184].

Modification by tyrosine nitration has been shown to impair the function of SERCA, reducing its ability to effectively transport Ca^2+^. For instance, nitration of residues Y294 and Y295 has been implicated in downregulating SERCA2 activity, thereby contributing to dysfunction in aging skeletal and cardiac muscle. Increased levels of nitration can correlate with various pathological states, including heart failure, since compromised SERCA function can lead to impaired calcium handling, ultimately contributing to cardiac contractile dysfunction [185]. Moreover, the vulnerability of SERCA to oxidative and nitrative stress is well documented, as multiple studies have shown that its critical role in calcium transport renders the enzyme highly susceptible to redox-dependent modifications under conditions of elevated ROS and RNS [186]. Notably, tyrosine nitration of SERCA impairs its Ca^2+^-ATPase activity, leading to reduced Ca^2+^ reuptake into the sarcoplasmic reticulum and elevated cytosolic Ca^2+^ levels, thereby contributing to cellular damage and cardiac dysfunction [187,188].

### 5.2 Pulmonary disease

#### 5.2.1 Activation of RhoA by nitration at tyrosine 34 drives endothelial dysfunction

Ras homolog family member A (RhoA) is a member of the small GTPase family that functions as a key regulator of the actin cytoskeleton and plays a central role in controlling endothelial barrier integrity. RhoA activation increases isometric tension at the cell membrane and promotes myosin-driven contractility, leading to the destabilization of endothelial junctions and increased vascular permeability [37]. RhoA acts upstream of Rho-associated kinase (ROCK), a major effector that drives cytoskeletal remodeling, stress fiber formation, and contractile responses in endothelial cells; through this pathway, RhoA contributes directly to barrier disruption during inflammatory lung injury [189]. The protein cycles between an inactive GDP-bound state and an active GTP-bound state, with this nucleotide exchange regulated by guanine nucleotide exchange factors (GEFs) that promote GDP dissociation, thereby controlling RhoA capacity to regulate cytoskeletal dynamics [190]. Because of its central role in endothelial tension, cytoskeleton organization, and barrier maintenance, RhoA is a critical molecular mediator linking inflammatory stimuli to downstream signaling events that drive endothelial dysfunction, ROCK activation, and mitochondrial fission during ALI [37,189].

RhoA undergoes selective nitration at Y34 in conditions of inflammatory lung injury. Tyrosine 34 is located within the switch I region adjacent to the nucleotide-binding site, and this modification has direct functional consequences for GTPase activation. Mass spectrometry identified Y34 as the single nitration site, and structural analyses demonstrated that nitration at this position induces a GEF-like conformational change that reduces GDP binding affinity, thereby stimulating RhoA activity and driving endothelial barrier disruption [37]. As shown in Fig. 9, this nitration-dependent activation of RhoA plays a central role in endothelial dysfunction during inflammatory lung injury. In human lung microvascular endothelial cells (HLMVEC), lipopolysaccharide (LPS) exposure increases RhoA nitration, thereby promoting activation of ROCK. Activated ROCK induces endothelial nitric oxide synthase (eNOS) uncoupling by driving its phosphorylation at T495, facilitating mitochondrial localization. This process enhances mitochondrial reactive oxygen species (ROS) production, leading to inflammasome activation and ultimately amplifying the inflammatory response [191]. In pulmonary arterial endothelial cells (PAECs) from a lamb model of pulmonary hypertension (PH), nitrated RhoA promotes a Warburg-like metabolic reprogramming. Site-specific nitration of RhoA at Y34 is required for downstream ROCK-dependent phosphorylation of dynamin-related protein 1 (Drp1) at S616, driving mitochondrial fission and increased mitochondrial reactive oxygen species (ROS) production. The resulting oxidative environment leads to cysteine oxidation and inhibition of prolyl hydroxylase domain protein 2 (PHD2), thereby stabilizing hypoxia-inducible factor-1α (HIF-1α) and enhancing glycolytic flux [192]. Collectively, these events provide a mechanistic basis for the hyperproliferative endothelial phenotype characteristic of PH [193,194].

**Fig. 9:**
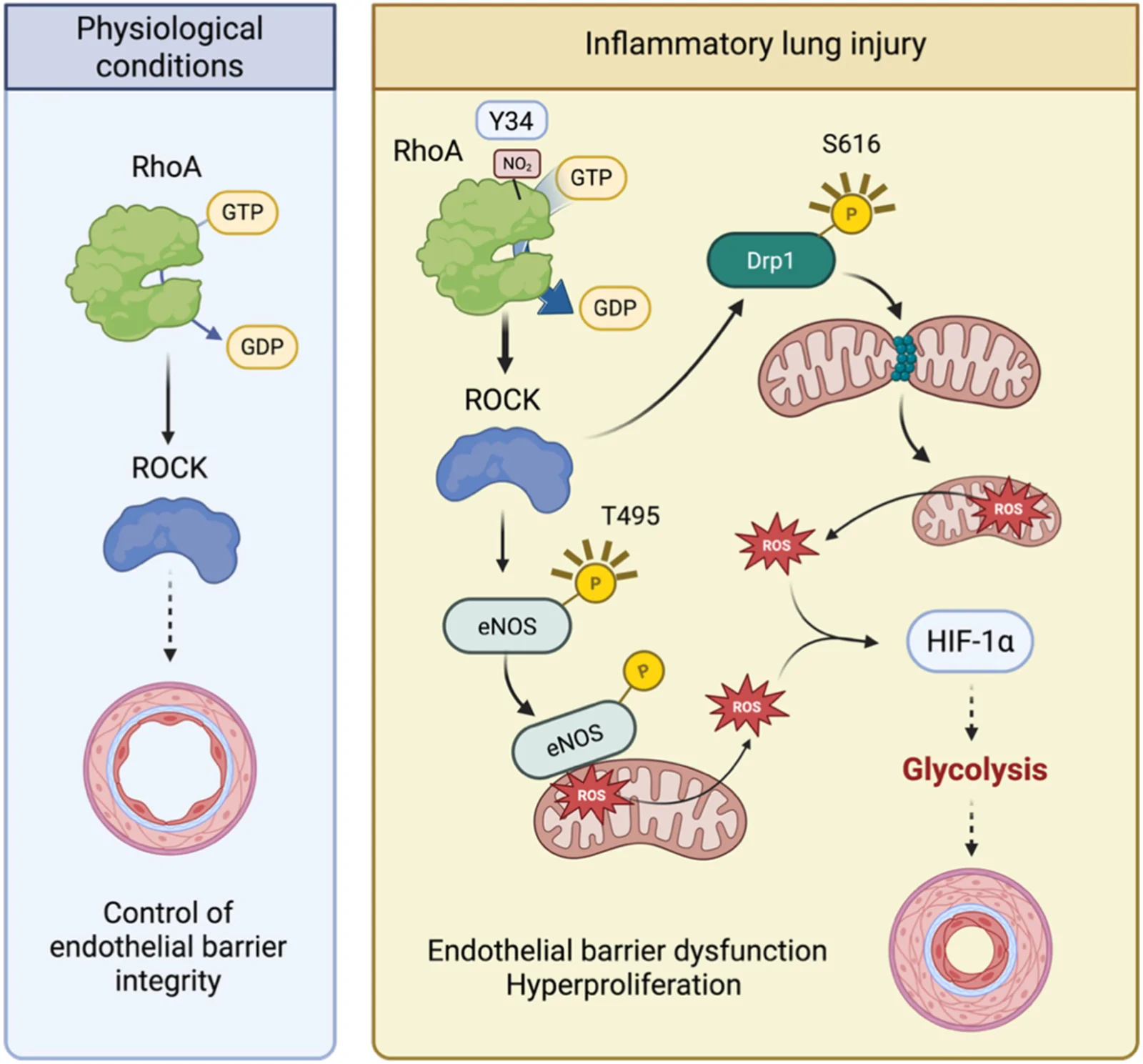
**Nitration-dependent activation of RhoA drives endothelial dysfunction during inflammatory lung injury.** Under physiological conditions (left), RhoA cycles between GDP- and GTP-bound states, maintaining controlled ROCK signaling and preserving endothelial barrier integrity. During inflammatory lung injury (right), RhoA undergoes selective tyrosine nitration at Y34, promoting a GEF-like conformational change that enhances GTP loading and sustained activation of RhoA. Activated RhoA stimulates ROCK signaling, leading to phosphorylation of endothelial nitric oxide synthase (eNOS) at T495, eNOS uncoupling and translocation to mitochondria, leading to increased mitochondrial reactive oxygen species (ROS) production. In parallel, ROCK-dependent phosphorylation of dynamin-related protein 1 (Drp1) at Ser616 induces mitochondrial fission and further elevates ROS levels. The resulting oxidative milieu stabilizes hypoxia-inducible factor-1α (HIF-1α), enhances glycolytic flux, and promotes a hyperproliferative endothelial phenotype. Collectively, these pathways converge to disrupt endothelial barrier function and amplify inflammatory signaling in lung injury.

An exciting therapeutic opportunity emerges from targeting RhoA nitration. Blocking RhoA nitration using the Y34-shielding peptide NipR1 (nitration inhibitory peptide for RhoA-1) attenuates RhoA activation, preserves endothelial barrier integrity, and reduces lung inflammation both *in vitro* and *in vivo* [37,191,194]. These findings confirm that Y34 nitration is a critical regulatory event linking nitrative stress to cytoskeletal remodeling and mitochondrial dysfunction during inflammatory lung injury.

#### 5.2.2 Inhibition of Rac1 by nitration at Y32: disruption of endothelial barrier integrity and vascular permeability

Rac1 is another member of the Rho family of small GTPases that, together with RhoA, plays a critical role in various cellular processes, including regulating the actin cytoskeleton, cell migration, and cell signaling pathways. RhoA and Rac1 exert opposing effects on endothelial function: RhoA promotes a contractile phenotype that leads to endothelial barrier disruption, whereas Rac1 stabilizes intercellular junctions and enhances barrier integrity [195]. Similar to RhoA, Rac1 cycles between an active GTP-bound state and an inactive GDP-bound state, a process also tightly regulated by GEFs [196,197]. Once activated, Rac1 interacts with numerous effector proteins that mediate functions such as the formation of lamellipodia, which are vital for cell movement and maintaining cellular morphology. This is particularly important in endothelial cells, where the balance between Rac1 and RhoA activities maintains vascular barrier integrity and regulates cell-cell junctions [191,195,198].

Wang et al. demonstrated that Rac1 undergoes nitration at Y32, a modification that inhibits Rac1 activity, linked to the disruption of endothelial barrier function during inflammatory conditions, such as those induced by LPS in cultured HLMVEC cells, and in PAEC exposed to 3-morpholinosydnonimine (SIN-1), which decomposes into ^•^NO and O_2_^•-^ [191]. The presence of nitrated Rac1 can lead to compromised junctional integrity in endothelial cells, resulting in increased vascular permeability and contributing to pathologies such as ALI [191,198]. The impact of tyrosine nitration on Rac1 is multifaceted. By modifying the GTPase structure, nitration may hinder its ability to interact effectively with GEFs or its downstream effectors, thereby impairing its normal signaling functions. Considering that RhoA and Rac1 have opposite physiological roles in the control of endothelial barrier integrity, simultaneous nitration of RhoA (activation) and Rac1 (inhibition) would exacerbate the disruption of the endothelial barrier and vascular permeability [191]. Promisingly, the shielding peptide NipR2, that effectively prevents nitration of Rac1 at Y32 in a mouse model of ALI induced by the intratracheal instillation of LPS, restored Rac1 activity, and attenuated symptoms of ALI, including alveolar damage [191]. This has significant implications for various physiological and pathological conditions, including inflammatory responses in the lungs [196,199].

#### 5.2.3 PKG-1α nitration: uncoupling NO–cGMP signaling in vascular dysfunction

Protein kinase G-1α (PKG-1α) is a central downstream effector of the •NO-soluble guanylyl cyclase (sGC)-cGMP axis, supporting vascular homeostasis by promoting smooth muscle relaxation and anti-proliferative/contractile programs. In pulmonary artery smooth muscle cells, PKG-1α expression reduces proliferation and favors expression of contractile markers, consistent with its physiological role in maintaining a quiescent, contractile phenotype [49]. In PH and in pulmonary vascular cell models exposed to nitrative stress/peroxynitrite-generating conditions, however, PKG-1α becomes susceptible to nitration at Y247 and Y425, detected by mass spectrometry analysis [49,200]. Mutational studies show that Y247 is functionally critical, because replacement of Y247 with nitration-resistant phenylalanine preserves kinase activity under nitrating conditions whereas unmodified PKG-1α loses activity, and overexpression of this nitration-resistant mutant decreases the proliferation of pulmonary artery smooth muscle cells (PASMC) [49,200]. Mechanistically, nitration at Y247 reduces PKG-1α affinity for cGMP, uncoupling the kinase from upstream cGMP signaling even when cGMP is present, thereby weakening vasodilatory signaling and enabling proliferative/phenotypic switching responses in vascular smooth muscle. In lung inflammatory injury models, nitrative stress increases PKG-1α nitration (including at Y247) and is linked to impaired cyclic nucleotide signaling control. PKG-1α normally inhibits phosphodiesterase 3A (PDE3A) via phosphorylation at S654, which supports higher intracellular cAMP, whereas loss of PKG activity under nitrative conditions permits higher PDE3A activity and reduced cAMP, contributing to endothelial barrier vulnerability [49,200] (Fig. 10). Together, these findings position PKG-1α nitration as a mechanistic example of how tyrosine nitration can convert a protective •NO-cGMP pathway into maladaptive vascular remodeling and lung dysfunction.

**Fig. 10:**
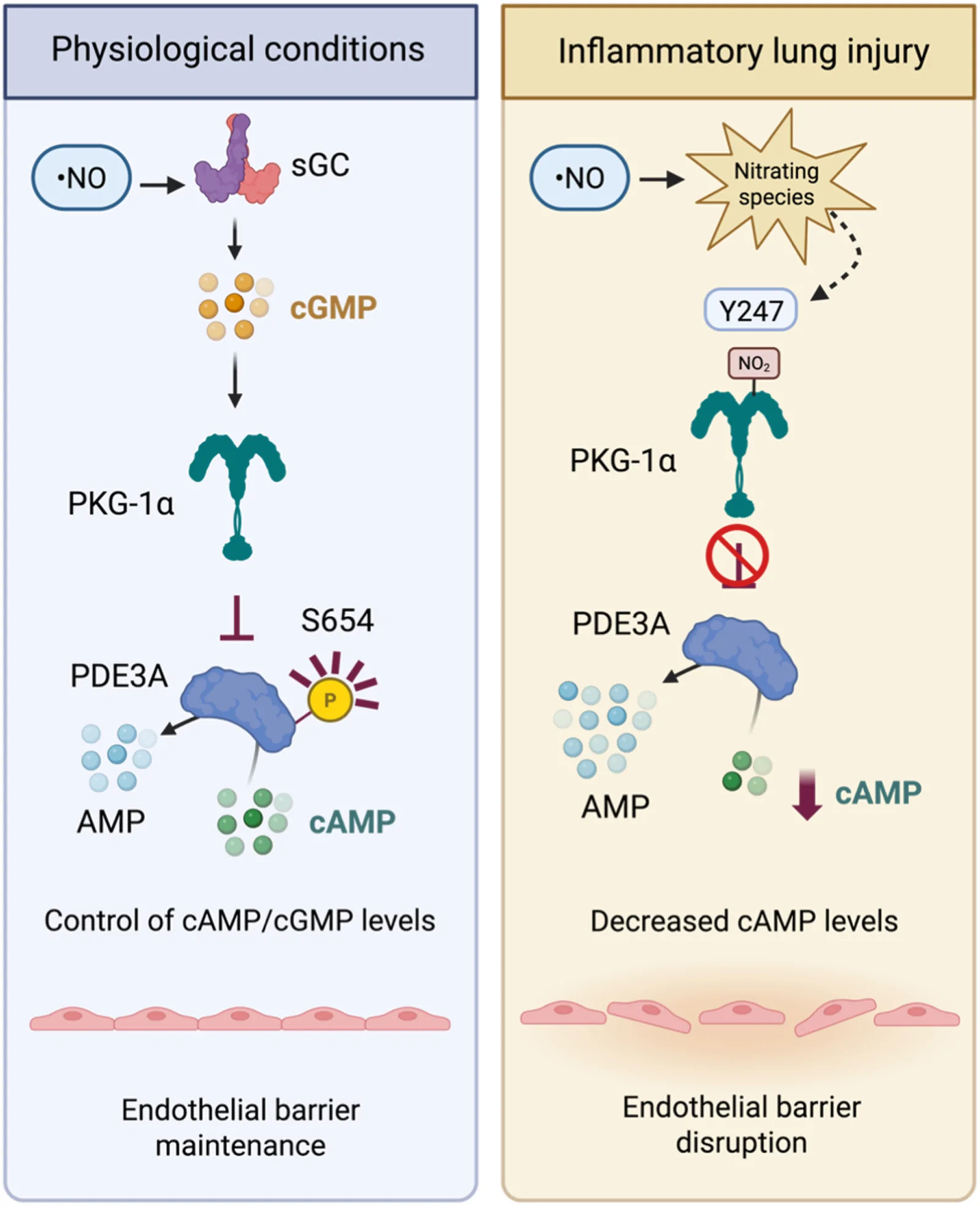
**Nitration of PKG1-a and the dysregulation of cAMP/cGMP signaling during inflammatory lung injury.** Under physiological conditions (left), nitric oxide (·NO) activates soluble guanylate cyclase (sGC), leading to cGMP production and activation of protein kinase G-1α (PKG-1α). PKG-1α phosphorylates phosphodiesterase 3A (PDE3A) at S654, inhibiting its activity and maintaining balanced cAMP/cGMP levels that support endothelial barrier integrity. During inflammatory lung injury (right), increased formation of nitrating species promotes tyrosine nitration of PKG-1α at Y247, impairing its kinase activity. Loss of PKG-1α signaling reduces inhibitory phosphorylation of PDE3A, resulting in enhanced cAMP hydrolysis and decreased intracellular cAMP levels. This disruption of cyclic nucleotide signaling compromises endothelial barrier function and contributes to vascular leak and inflammation.

In diseases characterized by acute and chronic inflammatory lung injury, including COPD, ARDS, and ALI, which can also progress to PH, the coordinated nitration of key signaling proteins such as RhoA, Rac1, and PKG1-α is likely to exert a profound influence on disease progression by reshaping endothelial signaling, barrier integrity, and inflammatory responses.

#### 5.2.4 Oxidative and nitrative regulation of HDAC2 activity

Histone deacetylase 2 (HDAC2) is a class I histone deacetylase that regulates chromatin structure and gene expression by removing acetyl groups from histones and other proteins. In the lung, HDAC2 is an important suppressor of inflammatory gene expression, and reduced HDAC2 activity has been linked to persistent inflammation and corticosteroid insensitivity in chronic obstructive pulmonary disease (COPD) [201].

Tyrosine nitration of HDAC2 is a selective and functionally disruptive post-translational modification. In peptide-mapping and cellular studies, peroxynitrite exposure induced nitration at multiple HDAC2 tyrosine residues, including Y68, Y73, Y146, Y167, Y173, and Y253, while Y238 was relatively resistant to nitration [50]. Importantly, these effects were examined in A549 human alveolar epithelial cells exposed to peroxynitrite, hydrogen peroxide, and cigarette smoke-conditioned medium, providing a defined oxidative/nitrative stress model [50]. Osoata et al. further showed that nitration at Y253 contributes to proteasomal degradation of HDAC2, since substitution of Y253 with alanine reduced HDAC2 degradation under these exposure conditions [50]. In contrast, nitration at Y146, located within a functional region of HDAC2, was associated with reduced HDAC2 enzymatic activity during early nitrative signaling [50]. Moreover, nitrated HDAC2 was detected in peripheral lung tissue from smokers and patients with COPD, supporting the *in vivo* relevance of this modification in chronic airway disease [50,201]. Collectively, these findings indicate that HDAC2 nitration is residue-specific and mechanistically consequential, promoting both loss of catalytic activity and enhanced degradation, thereby amplifying inflammatory responses in COPD.

## 6 Discussion

Protein tyrosine nitration has emerged as a pivotal post-translational modification that reshapes protein function and signaling networks and contributes directly to disease pathogenesis across multiple organ systems. Accumulating evidence from neurodegenerative, cardiovascular, and pulmonary disorders demonstrates that nitration is not a stochastic byproduct of oxidative stress, but rather a highly selective process that targets key regulatory proteins involved in metabolism, cytoskeletal organization, redox homeostasis, and stress signaling. This selectivity, driven by peroxynitrite and related reactive nitrogen species within inflammatory microenvironments, underscores nitration as a regulated and biologically meaningful modification, opening a novel mechanistic landscape that could be therapeutically exploited.

In neurodegenerative diseases such as Alzheimer's disease, Parkinson's disease, and ALS, nitration of proteins including GAPDH, α-synuclein, tau, Hsp70, and Hsp90 has been linked to protein misfolding and aggregation, mitochondrial dysfunction, impaired proteostasis, and progressive neuronal injury. Likewise, in cardiovascular and pulmonary pathologies, nitration of ApoA-I, RhoA, Rac1, and PKG-1α disrupts endothelial signaling, compromises barrier integrity, promotes inflammation, and drives maladaptive tissue remodeling. Importantly, crosstalk between tyrosine nitration and other post-translational modifications, most notably phosphorylation, adds an additional layer of complexity, enabling nitration to reprogram signaling pathways (Fig. 11).

**Fig. 11:**
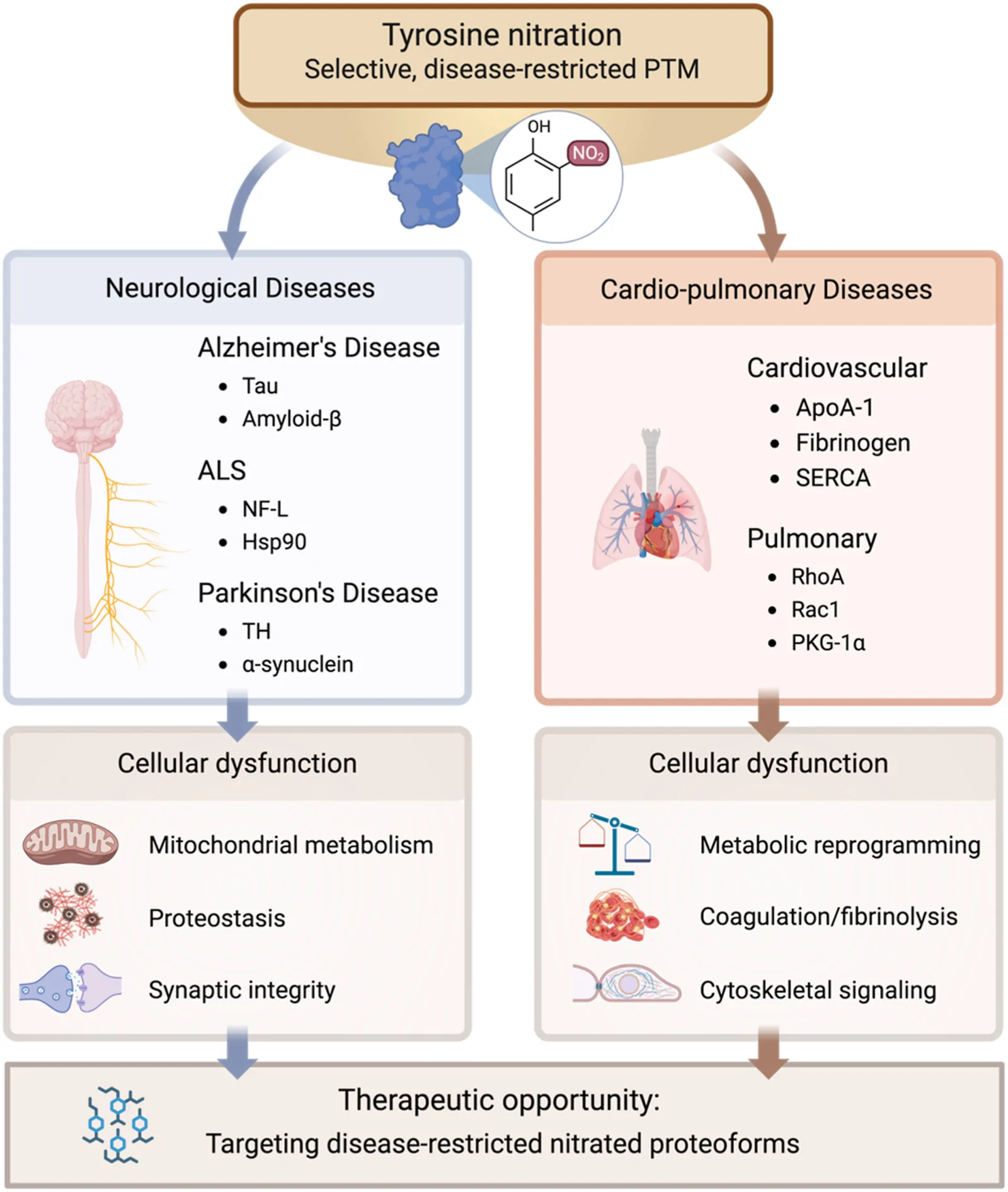
**Disease-associated protein tyrosine nitration across neurological and cardiopulmonary disorders.** Schematic overview of proteins reported to undergo tyrosine nitration in major neurological (left) and cardiopulmonary (right) diseases, and the cellular consequences. Protein tyrosine nitration, primarily driven by peroxynitrite and other RNS, selectively targets functionally critical tyrosine residues rather than occurring randomly. In neurological diseases, including Alzheimer's disease, amyotrophic lateral sclerosis (ALS), and Parkinson's disease, nitration preferentially affects proteins involved in cytoskeletal organization, proteostasis, mitochondrial metabolism, and neurotransmission, such as Tau, amyloid-β, neurofilament light chain (NF-L), Hsp90, tyrosine hydroxylase (TH), and α-synuclein. In cardio-pulmonary diseases, selective nitration targets proteins regulating lipid transport, coagulation and fibrinolysis, calcium handling, metabolic signaling, and cytoskeletal dynamics, including ApoA-1, fibrinogen, SERCA, RhoA, Rac1, and PKG-1α. These examples illustrate that tyrosine nitration is selective and context-dependent, reprogramming protein function and signaling networks in disease states, also highlighting the emerging therapeutic potential of targeting disease-restricted nitrated proteoforms.

As nitrated proteins emerge as active regulators rather than passive markers of disease, a deeper mechanistic understanding of the consequences of protein nitration, encompassing site-specific targeting, structural and conformational effects, downstream signaling outcomes, and interplay with other post-translational modifications could move the field toward the identification of novel therapeutic targets and shared pathogenic principles that transcend individual diseases. Such insights may facilitate the development of targeted therapeutic strategies designed to prevent, neutralize, or reverse nitration-driven dysfunction before irreversible damage occurs. Moreover, elucidating early nitration events could improve detection of early disease stages and inform precision interventions tailored to disrupt common redox-dependent mechanisms of progression. Table 2 summarizes the evidence currently supporting a pathogenic role for individual nitrated proteins in neurodegenerative and cardiopulmonary disease, distinguishing observations made in cellular and cell-free systems from those substantiated in animal models or human tissue. In addition, tyrosine nitration may coincide with other oxidative post-translational modifications on the same protein. To capture this, the table lists, for each protein, the additional oxidative post-translational modifications reported at the same or adjacent residues together with their described functional consequences. Viewed collectively, these entries highlight the recurrence of a limited set of nitrated targets across otherwise distinct pathologies, consistent with the shared pathogenic principles outlined above, as well as the frequent coexistence of several oxidative modifications on a single protein, whose functional outcomes may be convergent, additive or opposing. Table 2 thus provides both a survey of what is presently established and an indication of where site-specific, stoichiometry-controlled approaches are most needed.

**Table 2:** **Site-specific protein tyrosine nitration across neurological, cardiovascular, and pulmonary disease.** Proteins are grouped by organ system and disease. “Effect of nitration” indicates whether the modification causes loss-of-function, gain-of-function, or remains functionally uncharacterized. “Biological system” summarizes the experimental system in which nitration, or its functional consequences were tested (isolated protein *in vitro*, cultured cells with or without exogenous nitrating agents, or proteomic dataset). “*In vivo* evidence” states whether nitration of the protein has been demonstrated in human or clinical specimens or in animal models and identifies the system; where no such evidence has been reported, this is stated. Other oxidative and redox post-translational modifications are listed separately from their functional consequences. These modifications are compiled from the available literature and were not necessarily characterized in the same disease, tissue or biological setting as the nitration data; the system in which each was studied is indicated, and co-occurrence with tyrosine nitration on the same protein molecule is not implied. PTM: post-translational modification.

Protein (gene)	nY sites	Effect of nitration/pathophysiological consequence	Biological system	*In vivo*/clinical evidence	Evidence for impact on protein function	Other oxidative PTMs	Functional consequence of other PTM(s)	Ref.
NEURODEGENERATIVE - Alzheimer's disease								
Tau (MAPT)	Y18 Y29 Y197 Y394	**Site- and context-dependent effects:** nitration alters tau assembly and microtubule-related function. Site-specific nitrated tau species show distinct pathological distributions in AD tissue; direct effects on filament formation have mainly been shown experimentally.	*In vitro* nitration of recombinant tau.	**Yes-** human AD brain tissue (site-specific IHC: nY18 glial, nY29 neuronal NFTs).	Tau mutants containing one Y at position 18, 29, 197, or 394 and all other Y replaced with Phe were treated with ONOO^−^ followed by *in vitro* assembly assays.	C291 and C322 thiol oxidation with intra- and intermolecular disulfide formation; dityrosine cross-linking reported in AD-derived tau oligomers and paired helical filaments from patients.	*In vitro* models, disulfide-linked tau dimers promote conformational change, seeding and fibrillization; intramolecular C291-C322 linkage directs a structurally distinct amyloid polymorph. C322 lies within the fibril core of tau filaments solved from AD brain. Isolated four-repeat tau monomers with intramolecular disulfide bonds were able to form fibrils capable of self-seeding by recruiting oxidized monomers *in vitro*. Dityrosine cross-linking stabilizes AD-derived tau oligomers and paired helical filament/PHF-core structures.	[[68], [69], [70], [71], [72],[202], [203], [204]]
Amyloid-beta (APP/Aβ)	Y10	**Stabilization of oligomeric species:** Y10 nitration stabilizes oligomeric Aβ assemblies and enhances NMDAR-mediated excitotoxic signaling and synaptic dysfunction in experimental systems.	*In vitro*/cell-based Aβ oligomer studies.	**Yes-** human AD brain evidence for 3-nitrotyrosine/oxidative stress.	Aβ was nitrated *in vitro* using the peroxynitrite donor SIN-1. Nitration at the only Y residue in Aβ (Y10) was confirmed, and effects on aggregation were assessed *in vitro*. Neuronal toxicity was evaluated in hippocampal neurons.	M35 oxidation (sulfoxide); dityrosine cross-linking at Y10 (metal/copper-catalyzed ortho–ortho coupling).	*In vitro*, M35 oxidation to methionine sulfoxide slows Aβ_(1-42)_ fibrillization; consistent with this, other studies *in vitro* and cell-culture systems report that the resulting delay in aggregation is accompanied by reduced toxicity. However, when assessed at longer incubation times, Aβ_(1-42)_ shows toxicity *in vitro* and *in vivo*. Y10 dityrosine stabilizes dimers, oligomers and fibrils and is detected in amyloid plaques and CSF from AD patients (disease-matched).	[[80], [81], [82],[205], [206], [207], [208], [209], [210]]
ATP synthase alpha (ATP5F1A)	Y337 Y440	**Functionally uncharacterized/predicted loss:** nitration sites lie in the F1 catalytic domain; direct site-specific effects remain to be shown.	Redox-proteomic site identification; AD mitochondrial dysfunction context.	**Not shown-** site IDs from proteomic datasets only.	Y337/Y440 were identified as nitration sites by MS analysis, but their individual functional effects have not been tested experimentally.	C294 S-glutathionylation, S-nitrosylation/S-nitrosation, and C294-containing disulfide/Cysteine cross-linking; conserved C244/C294 reversible thiol oxidation also reported. These were mainly characterized in cardiac/mitochondrial and developmental models, not specifically in AD.	C294 acts as a redox-sensitive switch. S-glutathionylation and disulfide/cysteine cross-linking are associated with reduced ATP synthase/ATPase activity and mitochondrial dysfunction, whereas S-nitrosylation may displace inhibitory cysteine cross-linking and help restore ATP synthase activity in cardiac resynchronization therapy models. Reversible ATP-α-F1 oxidation at C244/C294 is also associated with impaired enzyme activity.	[13,[83], [84], [85],^88^,^89^,[211], [212], [213], [214]]
GAPDH	Y140 Y276 Y314 Y320	**Multiple reported consequences:** reduced metabolic function, altered protein interactions/localization, and aggregation-associated effects.	Redox-proteomic site identification across human AD brain, human COPD and aging skeletal muscle, and *in vitro* peroxynitrite-treated GAPDH.	**Yes-** nitrated GAPDH identified in human AD brain by redox proteomics; site-specific functional consequences not established.	Y140/Y276/Y314/Y320 identified as nitration sites by MS analysis but their individual functional effects have not been tested experimentally.	Catalytic C152 is the redox-sensitive cysteine switch in GAPDH (with C156). C152 undergoes S-nitrosylation, S-glutathionylation, and reversible oxidation to sulfenic acid, with irreversible hyperoxidation to sulfinic/sulfonic acid.	C152 oxidation inactivates the enzyme and reroutes glucose flux to the oxidative pentose phosphate pathway, increasing reductive capacity (characterized in tumor cells and mouse models, not in AD brain). S-nitrosylation inhibits glycolytic activity and augments pathological tau acetylation in the presence of Aβ, and S-glutathionylated GAPDH is elevated in blood with AD progression (both disease-matched).	[13,62,[91], [92], [93], [94], [95], [96], [97],[215], [216], [217]]
Hsp70 (HSPA1A)	Y336	**Functionally uncharacterized/predicted loss:** nitration detected, but direct effects on Hsp70 chaperone activity remain hypothetical.	MS dataset; endogenous nitration in peroxynitrite-treated PC12 cells (exogenous ONOO^−^).	**Not shown-** evidence limited to exogenous ONOO^−^ cell model and proteomic site data.	Y336 identified as a nitration site by MS analysis, but the functional consequence of nitration at this site has not been tested experimentally.	Cysteine redox PTMs of HspA1A/Hsp70, including S-glutathionylation at C574 and C603 in the C-terminal α-helical lid; broader cysteine redox modifications including S-nitrosylation and S-sulfhydration have also been reported. These were characterized mainly in redox/cell models, not specifically in AD.	S-glutathionylation of C574/C603 promotes unfolding of the C-terminal α-helical lid, allowing the lid region to occupy the substrate-binding site and reduce binding of external client proteins. This suggests redox-dependent regulation of Hsp70 substrate binding/chaperone function, but AD-specific consequences remain unresolved.	[13,34,[98], [99], [100],[218], [219], [220]]
MnSOD (SOD2)	Y34	**Loss-of-function:** reduced dismutase activity, impaired antioxidant defense, mitochondrial dysfunction, and feedforward oxidative/nitrative stress.	Classic *in vitro* functional nitration studies.	**Yes-** disease association in AD models/tissue context.	Incorporation of site-specific nitrotyrosine by genetic code expansion (GCE) at position 34 impaired MnSOD enzymatic activity.	C196 redox modification, including S-glutathionylation; reported mainly in biochemical/mitochondrial redox contexts, not specifically in AD.	Functional consequence is less clearly defined than Y34 nitration. Cysteine redox modification may reflect redox-sensitive regulation or protection from irreversible thiol oxidation, but direct effects on MnSOD activity in AD remain unclear.	[18,[101], [102], [103], [104], [105],^107^,[221], [222], [223]]
**NEURODEGENERATIVE - Amyotrophic lateral sclerosis (ALS)**								
Neurofilament-L (NEFL)	Y17, Y138 Y177 Y265 Y372	**Decreased assembly *in vitro*** (Y17, Y138, Y177, Y265)**,** and functionally uncharacterized (Y372). Nitrated NF-L is associated with ALS pathology; proposed effects include altered filament assembly and axonal organization.	*In vitro* and cell studies.	**Yes-** human ALS spinal cord (nitrated NF-L accumulation; colocalizes with nitrotyrosine); Y372-specific consequences remain inferred.	*In vitro* ONOO^−^ treatment in the presence of SOD1 with nitration sites identified by MS analysis. Nitration correlated with decreased neurofilament assembly. Nitration at Y372 was identified by MS analysis in a dataset from A549 cell lysates. The sites were not selectively manipulated in functional studies.	Oxidative NF-L aggregation with dityrosine cross-linking; protein carbonyl formation also reported in catechol-neurotoxin models. Sites are not clearly resolved. These modifications were characterized mainly *in vitro*/toxin-induced oxidative-stress models, not specifically as site-mapped ALS modifications.	Oxidative cross-linking promotes NF-L aggregation and may disturb neurofilament assembly and axonal cytoskeletal organization. Acrolein-mediated NF-L aggregation shows amyloid-like features, but direct disease-specific consequences in ALS remain unresolved.	[13,48,97,[117], [118], [119], [120],[224], [225], [226]]
Hsp90 (HSP90AA1/HSP90AB1)	**Y33** **Y56** Y276 Y484 Y596	**Gain-of-function:** Hsp90-nY56 activates P2X7R/Ca^2+^ influx/Fas-dependent apoptosis in motor neurons; Hsp90-nY33 downregulates mitochondrial metabolism; nitration at Y33 or Y56 differentially affects Hsp90 structure.	Mechanistic *in vitro* and cell studies.	**Yes-** endogenous Hsp90-nY56 in human ALS motor neurons; G93A and DCTN1 mouse models.	*In vitro* ONOO^−^treatment led to identification of Y33, Y56, Y276, Y484, and Y596 as nitration sites by MS analysis. Sites were individually and/or collectively replaced with Phe (to assess necessity), or nitrotyrosine incorporated site-specifically using GCE (to assess sufficiency), determining functional impact on structure, function, and biological activity in cells.	C597 S-nitrosylation (C-terminal domain, eNOS-interacting region); S-glutathionylation at C366 and C412 (middle domain).	In endothelial cells, S-nitrosylation of C597 inhibits Hsp90 ATPase activity, alters dimerization and abolishes its positive regulation of eNOS, forming a negative-feedback loop limiting NO synthesis. S-glutathionylation at C366/C412 (characterized in breast cancer cells) abolishes ATPase activity and enhances Hsp90 degradation. Neither modification has been examined in ALS motor neurons.	[34,35,[121], [122], [123], [124], [125],[227], [228], [229]]
**NEURODEGENERATIVE - Parkinson's disease (PD)**								
alpha-Synuclein (SNCA)	Y39 Y125 Y133 Y136	**Aggregation:** nitration promotes aggregation/fibrillation, toxic oligomers, impaired synaptic function, and neuronal vulnerability; GLOD4 can catalyze selective alpha-synuclein nitration.	Cell and *in vitro* aggregation studies.	**Yes-** human PD/synucleinopathy post-mortem tissue; *in vivo* mouse GLOD4/methamphetamine model.	Recombinant α-synuclein treated with ONOO^−^/CO_2_ or MPO/H_2_O_2_/nitrite followed by aggregation/polymerization analyses. Disease-related functional causality has not been established individually for the four sites.	Methionine oxidation at M1, M5, M116 and M127 (sulfoxide); Dityrosine cross-linking involving α-synuclein tyrosine residues, with Y39 and C-terminal tyrosines implicated in different experimental systems.	Alpha-synuclein lacks cysteine residues, but methionine oxidation at different positions has been reported. *In vitro* and in cell models, methionine oxidation inhibits fibrillization and stabilizes soluble oligomers; N-terminal M1/M5 sulfoxide are repaired by methionine sulfoxide reductase whereas C-terminal M116/M127 persist and impair Fyn-mediated Y125 phosphorylation, a nitration site in this table. Dopamine-mediated M127 oxidation causes oligomerization and cytotoxicity in catecholaminergic PC12 cells. Dityrosine cross-linking stabilizes oligomers; reports differ on whether it promotes or inhibits fibril formation.	[32,61,[126], [127], [128], [129], [130], [131], [132],[135], [136], [137], [138],[230], [231], [232], [233], [234], [235], [236]]
Tyrosine hydroxylase (TH)	Y423 Y428 Y432	**Loss-of-function:** reduced catalytic activity and impaired dopamine biosynthesis.	*In vitro* ONOO^−^/•NO_2_ exposure with activity loss.	**Not shown-** PD relevance mechanistically inferred.	Y423/Y428/Y432 identified as nitration sites by MS analysis, followed by single, double, and triple positional scanning performed by replacement with Phe and ONOO^−^ treatment *in vitro*. Functional effects are not explained by nitration of one individual site.	Cysteine redox modifications, including S-glutathionylation of rat TH at C177, C249, C263, C329, C330 and C380; S-nitrosylation at C279; and dopamine-oxide/quinone modification of human TH at C176. These were characterized mainly in purified enzyme, PC12/cell, and mouse striatal models, not as PD tissue-mapped oxidative PTMs.	S-glutathionylation inhibits TH catalytic activity and can be reversed by reducing systems such as DTT/glutaredoxin. Dopamine-oxide modification of C176 also inhibits TH activity. In contrast, S-nitrosylation at C279 increases TH enzymatic activity by increasing Vmax, showing that different cysteine redox PTMs can have opposite effects on TH function.	[133,134,[139], [140], [141], [142],[237], [238], [239]]
**CARDIOVASCULAR DISEASE - Thrombosis**								
Fibrinogen (FGA/FGB/FGG)	Bβ Y292 Y422	**Prothrombotic function:** accelerated clot formation, denser/stiffer fibrin networks, impaired fibrinolysis, and altered clot biomechanics.	*Ex vivo* studies of nitrated fibrinogen isolated from smokers and non-smokers individuals.	**Yes-** human plasma/tissue (smokers, CVD, VTE, ARDS).	Y292 and Y422 identified as nitration sites *in vivo* by MS analysis, followed by nitrated fibrinogen isolation (anti-nitrotyrosine antibodies) and *ex vivo* polymerization assays. The sites were not selectively manipulated in functional studies.	Methionine oxidation to methionine sulfoxide, especially Aα M476 and Bβ M367; broader oxidative modification/carbonylation and dityrosine cross-linking have also been reported. These were characterized mainly in purified fibrinogen, and in oxidative stress and thrombotic/plasma contexts.	Oxidation of fibrinogen alters fibrin polymerization and clot architecture. Oxidation of Aα M476 in the αC domain and Bβ M367 has been associated with reduced lateral aggregation, thinner fibrin fibers, altered clot mechanics, reduced permeability, and altered fibrinolysis. Carbonylation and broader oxidation are also associated with impaired fibrin monomer polymerization and abnormal clot structure.	[17,20,[143], [144], [145], [146], [147], [148], [149], [150],[156], [157], [158], [159],[240], [241], [242]]
**CARDIOVASCULAR DISEASE - Fibrinolysis**								
Plasmin/plasminogen (PLG)	N/R	**Loss-of-function:** reduced plasmin catalytic activity and impaired fibrinolysis, contributing to a prothrombotic state.	Functional *in vitro* studies with peroxynitrite/nitrative stress.	**Yes-** human plasma (elevated in smokers); site-specific nitration not resolved.	ONOO^−^ treatment *in vitro* was associated with increased nitration and reduced plasmin/fibrinolytic activity, but individual sites were not identified.	Oxidative modification of plasminogen by H_2_O_2_ or HOCl/OCl^−^, including methionine and tryptophan oxidation in different structural regions; specific disease-matched sites are not clearly established.	Oxidation of plasminogen decreases the fibrinogenolytic/fibrinolytic activity of the plasmin formed from oxidized plasminogen, supporting reduced clot-degrading capacity under oxidative stress.	[7,148,[151], [152], [153], [154], [155],^243^,^244^]
**CARDIOVASCULAR DISEASE - Atherosclerosis**								
ApoA-I	Y166 (HDL/artery wall), Y192 (lipid-free); Y18 Y29 Y115 Y236	**Loss-of-function:** Y166 nitration impairs LCAT activation and promotes dysfunctional HDL; Y192 has limited effect on ABCA1-mediated efflux.	*In vitro*/lipid-state analyses.	**Yes-** apoA-I from human atherosclerotic arteries/plaque; Y166 a major physiological nitration site.	Site-specific incorporation of nitrotyrosine at position 166 impaired LCAT activity *in vitro*. Y192 nitration was studied separately following MPO-dependent nitration.	MPO-dependent chlorination of Y192 (3-chlorotyrosine); methionine oxidation at M86, M112 and M148 (sulfoxide).	Y192 chlorination together with methionine oxidation impairs ABCA1-dependent cholesterol efflux by blocking apoA-I binding to ABCA1 and JAK2 signalling; Y192F substitution plus methionine sulfoxide reductase treatment restores efflux. Notably, chlorination but not nitration impairs the ABCA1 interaction. 3-chlorotyrosine is elevated in HDL from human atherosclerotic lesions (disease-matched).	[[160], [161], [162], [163], [164], [165], [166],^245^]
LDL/ApoB-100	Y276 Y666 Y720 Y2524 Y3139 Y3295 Y3489 Y4141	**Atherogenic function:** ApoB nitration associates with alpha-helix loss/beta-sheet gain, LDL unfolding, and increased uptake through LOX-1/CD36/SR-A pathways.	LC-MS/MS site identification; receptor-uptake studies.	**Yes-***in vivo*-modified LDL (LDL^−^) from human circulation with mapped nitrotyrosine sites.	Human LDL^−^ and LDL treated with ONOO^−^ or SIN-1 *in vitro* and nitration sites identified by MS analysis, but individual sites were not selectively manipulated.	**Yes-** in vivo-modified LDL (LDL^−^) from human circulation with mapped nitrotyrosine sites.	ApoB oxidation/lipoxidation alters LDL structure and receptor recognition, reducing normal LDL receptor handling and promoting scavenger-receptor-mediated macrophage uptake, foam-cell formation, endothelial dysfunction, and atherogenic inflammation.	[13,[168], [169], [170], [171], [172], [173], [174],^246^,^247^]
**CARDIOVASCULAR DISEASE - Atherosclerosis**								
COX-1 (PTGS1)	Y385 (catalytic, conserved)	**Loss-of-function:** Y385 nitration abolishes cyclooxygenase activity and may disrupt thromboxane/prostanoid balance.	Purified COX-1 + ONOO^−^ studies.	**Yes-** COX nitration detected in human atherosclerotic tissue and ApoE^−/−^ mouse lesions.	ONOO^−^ or SIN-1 treatment *in vitro* caused Y385 nitration and correlated with pronounced loss of COX-1 activity, but the site was not selectively manipulated in functional studies.	Peroxide-dependent oxidative inactivation of prostaglandin H synthase-1/COX-1 involving high-valent oxyferryl heme and enzyme radical intermediates; specific stable sidechain PTM sites are not clearly resolved.	Peroxide-driven oxidative inactivation reduces COX/peroxidase catalytic activity, suggesting that COX-1 function can be regulated or damaged by oxidative chemistry in addition to Y385 nitration. Disease-specific co-occurrence with COX-1 nitration remains unresolved.	[17,[177], [178], [179], [180], [181],^248^,^249^]
**CARDIOVASCULAR DISEASE - Cardiac dysfunction**								
SERCA2a (ATP2A2)	Y294, Y295	**Loss-of-function:** reduced Ca^2+^-ATPase activity, impaired SR Ca^2+^ reuptake, elevated cytosolic Ca^2+^, and contractile dysfunction.	Functional nitration studies.	**Yes-** disease correlation in heart failure/aging muscle.	Y294/Y295 nitration in aging skeletal/cardiac muscle by MS analysis and subsequently detected in tissues with antibodies raised against the di-nitrated Y294/Y295 peptide. Increased nitration of these residues correlated with impaired SERCA activity, but the sites were not selectively manipulated in functional studies.	C674 S-glutathionylation (reversible); irreversible oxidation of C674 to sulfonic acid; oxidation at C498 also reported.	In vascular smooth muscle, peroxynitrite-driven S-glutathionylation of C674 activates SERCA, increasing Ca^2+^ uptake to mediate •NO-dependent arterial relaxation; mutation of C674 by serine abolishes this effect. Irreversible sulfonic acid oxidation of C674 occurs in atherosclerosis and aortic aneurysm and blocks the activating modification; C498 oxidation promotes SERCA2a degradation in heart. Note this is the opposite direction to Y294/Y295 nitration and is characterized in vascular rather than failing myocardial tissue	[[182], [183], [184], [185], [186], [187], [188],^250^]
**PULMONARY DISEASE - Acute Lung Injury (ALI) & Pulmonary Hypertension (PH)**								
RhoA	Y34 (switch I domain)	**Activation:** GEF-like conformational change reduces GDP binding and activates RhoA/ROCK signaling, promoting barrier disruption, eNOS uncoupling, mitochondrial ROS, and metabolic reprogramming.	MS and structural studies; HLMVEC/LPS and pulmonary vascular cell models.	**Yes-** lamb PH PAEC model; NipR1 shielding peptide rescues *in vivo*.	Nitration at Y34 identified as a single nitration site by MS analysis. The NipR1 shielding peptide but not a Phe-containing peptide selectively reduced Y34 nitration, RhoA activation, and associated endothelial-barrier dysfunction *in vivo*.	Redox-sensitive C20 (and adjacent C16) in the GXXXCGK(S/T) C p-loop motif: thiol oxidation to sulfenic/sulfinic acid, S-glutathionylation and S-nitrosylation.	*In vitro* and in cell studies, oxidation of the p-loop thiol displaces the bound nucleotide and accelerates intrinsic nucleotide exchange (up to ∼1000-fold with radical oxidants), activating RhoA. S-nitrosylation, by contrast, attenuates RhoA activity, opposite to the activation triggered by Y34 nitration. Largely general GTPase biochemistry rather than ALI- or PH-specific.	[37,[189], [190], [191],^194^,^251^]
**PULMONARY DISEASE - ALI**								
Rac1	Y32	**Loss-of-function:** Rac1 inhibition compromises junctional integrity and increases vascular permeability in ALI models.	HLMVEC/LPS; PAEC/SIN-1.	**Yes-** NipR2 shielding-peptide rescue in mouse ALI model.	Nitration at Y32 identified by MS analysis in LPS-treated HLMVEC. The NipR2 shielding peptide but not a Phe-containing peptide selectively reduced Y32 nitration and preserved endothelial barrier function *in vivo*.	Redox-sensitive C18 in the GXXXXGK(S/T) C p-loop motif: thiol oxidation and S-glutathionylation; C81 and C157 also implicated.	S-glutathionylation at C18 increases nucleotide exchange ∼200-fold *in vitro*. In human aortic endothelial cells under hyperglycaemic/hyperlipidaemic stress, Rac1 glutathionylation is associated with inactivation and vascular barrier dysfunction and is reversed by glutaredoxin-1; endothelial, but in a metabolic-disease rather than LPS/ALI context.	[191,[195], [196], [197], [198], [199],^251^,^252^]
**PULMONARY DISEASE - PH**								
PKG-1alpha (PRKG1)	Y247, Y425	**Loss-of-function:** Y247 nitration reduces cGMP affinity and uncouples NO-cGMP signaling, reducing kinase activity and promoting proliferative vascular remodeling.	MS and mutational studies (Y247F protection); pulmonary vascular cells.	**Yes-** PH animal and cell models.	HEK-293T cells expressing PKG-1α were exposed to SIN-1. Y247 and Y425 were identified as nitration sites by MS analysis. Only replacement with Phe at position 247 preserved PKG-1α activity during nitrative stress.	C42 interprotein disulfide (homodimer, N-terminal leucine zipper); intraprotein disulfides involving C117 and C195 also reported.	C42 disulfide bond formation activates PKG-1α independently of NO–cGMP, increasing substrate affinity and driving vasodilation; ‘redox-dead’ C42S knock-in mice are hypertensive. cGMP binding prevents disulfide formation. This is the opposite direction to Y247 nitration and is characterized in the systemic circulation and blood-pressure control rather than in pulmonary hypertension.	[49,200,253,254]
**PULMONARY DISEASE - Chronic Obstructive Pulmonary Disease (COPD)**								
HDAC2	Y68, Y73, Y146, Y167, Y173, Y253 (Y238 resistant)	**Loss-of-function plus degradation:** Y146 reduces enzymatic activity; Y253 promotes proteasomal degradation, amplifying inflammation and corticosteroid insensitivity.	HDAC2 was treated with ONOO^−^*in vitro* and A549 cells exposed to ONOO^−^/H_2_O_2_/cigarette-smoke conditioned medium.	**Yes-** nitrated HDAC2 detected in human smoker COPD lung tissue.	Y68, Y73, Y146, Y167, Y173, and Y253 were identified as nitration sites by MS analysis. Tyr→Ala mutagenesis at position 253 showed that Y253 is at least partly responsible for nitrative-stress-induced HDAC2 proteasomal degradation.	S-nitrosylation at C262 and C274 (adjacent to the catalytic center).	In cortical neurons, S-nitrosylation does not alter deacetylase activity but induces HDAC2 release from chromatin, increasing histone acetylation and activating neurotrophin-dependent genes; the C262/274A mutant acts as a strong repressor. In skeletal muscle (Duchenne dystrophy models), S-nitrosylation instead decreases HDAC2 activity. In heart, S-nitrosylation of HDAC at C262/274 is associated with diastolic dysfunction. Not examined in COPD lung, where Y146/Y253 nitration is reported.	[50,201,[255], [256], [257]]

Protein tyrosine nitration displays disease-restricted patterns, functional specificity, and mechanistic impact, and on that basis holds considerable promise both as a biomarker of early pathology and as a tractable therapeutic target. Testing that promise has long been limited by the absence of tools to modulate nitration at defined sites.

That gap has begun to close with the development of genetic code expansion tools to express and purify site-specific proteins using bacterial models. Amber suppression in bacteria, using orthogonal aminoacyl-tRNA synthetase/tRNA pairs, allows 3-nitrotyrosine to be incorporated at any position specified by a TAG codon, yielding homogeneous, quantitatively nitrated protein for structural and functional analysis [15,34,121,123,124,223,[258], [259], [260], [261]]. Delivering such proteins into cells, as we have done in several mammalian systems, extends that control into the cellular context [34,35,121,123,124]. Delivering site-specifically nitrated protein in parallel with a control in which the target tyrosine is substituted by phenylalanine provides paired gain- and loss-of-function arms, resolving the contribution of nitration at single-residue resolution in disease-relevant culture models. Since phenylalanine substitution precludes phosphorylation as well as nitration, the loss-of-function arm reports the absence of both modifications; specificity is therefore established by the gain-of-function arm, in which 3-nitrotyrosine is present at a single defined position. Critically, the fraction of a given protein carrying the modification can first be quantified in diseased tissue and then reproduced in cellular models by titrating the delivered protein. Function is thus interrogated at a physiologically calibrated occupancy, converting the low and variable stoichiometry that confounds correlative studies into a defined experimental parameter.

The reach of these tools is nonetheless confined to short-term experiments: the delivered protein is cleared by degradation and dilution through cell division, so the accessible experimental window is set by the half-life of the protein under study. Methodologies allowing sustained expression of site-specifically nitrated proteins *in vivo* therefore remain an outstanding need, with a genetic code expansion system compatible with mammalian cell systems currently under development [260,261].

The methodologies now available to detect and mechanistically probe nitrated proteins, from immunochemical detection and mass-spectrometry-based site mapping to chemical enrichment, genetically encoded nitration and intracellular delivery, differ widely in sensitivity, specificity and technical demand. Their respective applications and complementarities warrant a dedicated methodological review. Collectively, these approaches mark a shift from detecting nitrated proteins to defining what nitration does at single-residue resolution. Extending that resolution to the proteome scale and to *in vivo* models is the principal challenge ahead.

Meeting that challenge will require tyrosine nitration to be treated not as an isolated modification but as one node within the wider redox proteome, interpreted alongside cysteine oxidation and other oxidative post-translational modifications, and alongside non-redox modifications at the same or neighboring residues, most notably phosphorylation. Integrating site-resolved nitration data into systems-level redox biology and clinical proteomics frameworks will ultimately be critical for translating these advances into diagnostic and therapeutic applications, whether as biomarkers for patient stratification or as defined residues amenable to pharmacological or genetic intervention. Together, these efforts position tyrosine nitration as a unifying lens through which novel redox-driven disease mechanisms can be understood and therapeutically exploited.

## CRediT authorship contribution statement

**Nirbachita Adrita:** Writing – review & editing, Writing – original draft, Investigation, Data curation, Conceptualization. **Inge Claassen:** Writing – review & editing. **Miranda Huerta:** Writing – review & editing. **Stephen M. Black:** Writing – review & editing. **Alvaro G. Estevez:** Writing – review & editing. **Blaine R. Roberts:** Writing – review & editing. **Joseph S. Beckman:** Writing – review & editing. **Maria Clara Franco:** Writing – review & editing, Visualization, Supervision, Resources, Project administration, Funding acquisition, Conceptualization.

## Declaration of competing interest

The authors have no competing interests.

## Data Availability

No data was used for the research described in the article.
